# A quantitative indicator diagram for lytic polysaccharide monooxygenases reveals the role of aromatic surface residues in *Hj*LPMO9A regioselectivity

**DOI:** 10.1371/journal.pone.0178446

**Published:** 2017-05-31

**Authors:** Barbara Danneels, Magali Tanghe, Henk-Jan Joosten, Thomas Gundinger, Oliver Spadiut, Ingeborg Stals, Tom Desmet

**Affiliations:** 1 Centre for Synthetic Biology (CSB), Department of Biochemical and Microbial Technology, Faculty of Bioscience Engineering, Ghent University, Ghent, Belgium; 2 Bio-Prodict BV, Nijmegen, The Netherlands; 3 Research Division Biochemical Engineering, Institute of Chemical Engineering, TU Wien, Vienna, Austria; 4 Industrial Catalysis and Adsorption Technology (INCAT), Faculty of Engineering and Architecture, University of Ghent, Ghent, Belgium; Institut National de la Recherche Agronomique, FRANCE

## Abstract

Lytic polysaccharide monooxygenases (LPMOs) have changed our understanding of lignocellulosic degradation dramatically over the last years. These metalloproteins catalyze oxidative cleavage of recalcitrant polysaccharides and can act on the C1 and/or C4 position of glycosidic bonds. Structural data have led to several hypotheses, but we are still a long way from reaching complete understanding of the factors that determine their divergent regioselectivity. Site-directed mutagenesis enables the investigation of structure-function relationship in enzymes and will be of major importance in unraveling this intriguing matter. In this context, it is crucial to have an enzyme assay or screening approach with a direct correlation with the desired functionality. LPMOs render this search extra challenging due to their insoluble substrates, complex pattern of reaction products and lack of synthetic standards of most oxidized products. Here, we describe a regioselectivity indicator diagram based on the time-course of only 2 HPAEC-PAD signals. The diagram was successfully used to confirm the hypothesis that aromatic surface residues influence the C1/C4 oxidation ratio in *Hypocrea jecorina* LPMO9A. Consequently, the diagram should become a valuable tool in the search towards better understanding and engineering of regioselectivity in LPMOs.

## Introduction

Since their boosting effect on biomass degradation was discovered in 2010 [1], insight in the mode of action of lytic polysaccharide monooxygenases (LPMOs) has gradually increased. These copper-dependent enzymes oxidatively cleave the glycosidic bonds of polysaccharides using molecular oxygen and an electron donor. Although first only considered to be active on chitin and cellulose, LPMOs active on hemicellulose, starch and soluble cello-oligosaccharides have recently been identified [2–5].

**Fig 1:**
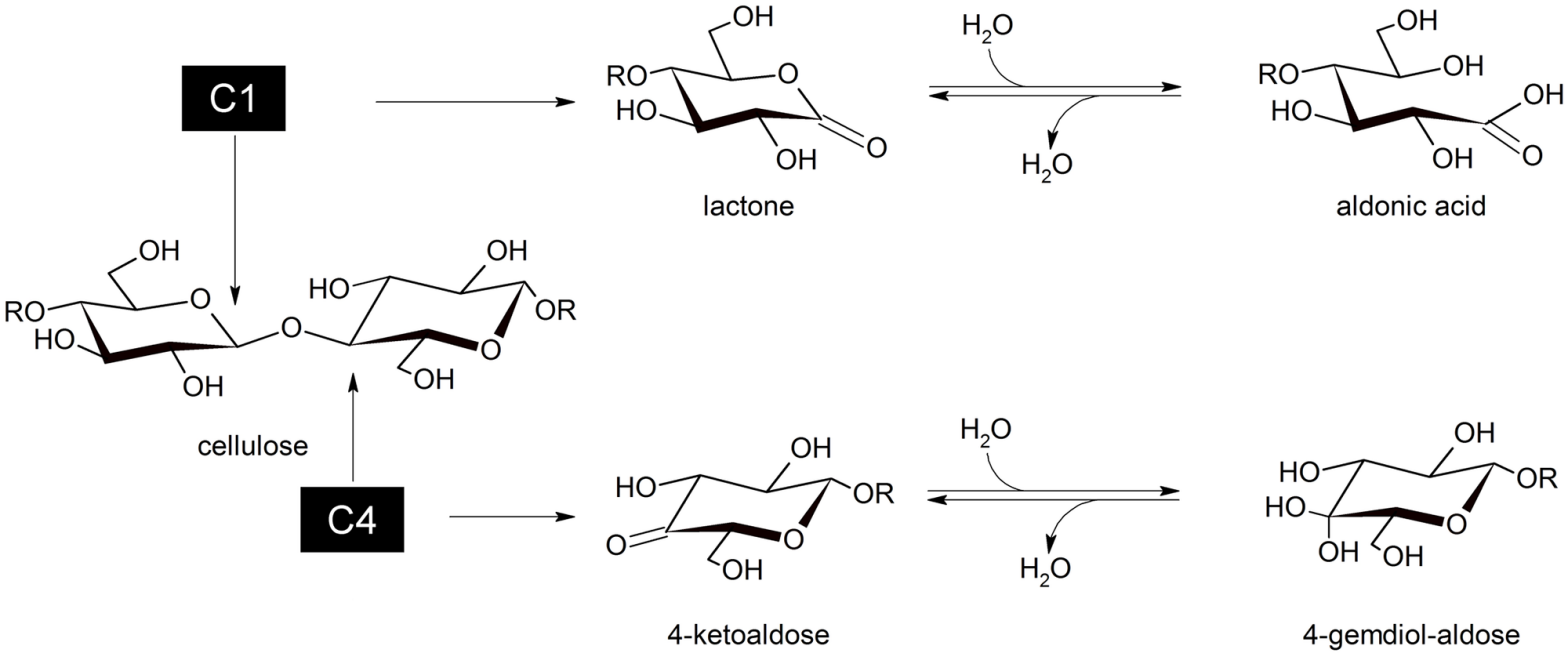
LPMO regioselectivity. Oxidation of the C1 position generates a lactone, which is hydrated to a reducing-end aldonic acid. C4-oxidation leads to non-reducing-end 4-ketoaldose formation, which will spontaneously hydrate to gemdiols in aqueous conditions.

In the Carbohydrate Active Enzyme database (CAZy), LPMOs are currently classified in Auxiliary Activity (AA) family 9, 10, 11 and 13 [4,6,7]. LPMOs all share a beta-sandwich fold with a planar surface with several aromatic and polar residues forming a polysaccharide binding, CBM-like structure [8–12]. On this planar surface, a copper ion ligated by a ‘histidine brace’ is responsible for the oxidative action [13,14]. Although the conserved active site architecture suggests a similar reaction mechanism, different LPMOs can oxidize the C1 and/or C4 position, generating aldonic acids and 4-ketoaldoses respectively (Fig 1). C6-oxidation has also been suggested for *Ta*LPMO9A [13] and *Pa*LPMO9B [15], but the formation of 6-hexodialdoses is under debate as their molecular weight is the same as for 4-ketoaldoses and C6-oxidation does not actually result in chain cleavage [16].

Several hypotheses have been formulated on the underlying causes for these differences in regioselectivity. Based on the accumulating structural data, the accessibility of the solvent-facing axial position of the catalytic copper site is believed to determine the C1/C4-oxidation ratio [17,18]. Structural features of the planar surface have also been investigated. A subdomain of about 14 residues was shown to be important for C4 oxidation in *Neurospora crassa* LPMOs [19]. The positioning of N-glycans and aromatic residues has also been suggested to be distinctive [9,10,12,20,21]. And very recently, a shift in released products was observed after carbohydrate binding module (CBM) removal and fusion, implying that in LPMOs, CBMs might not only play a role in substrate binding but also in regioselectivity [22]. It is clear that mutagenesis studies are needed to further elucidate the structural features contributing to regioselectivity.

Except for a colorimetric assay to test the catalytic efficiency based on a side reaction of LPMOs [23], all present studies are based on lengthy incubations with the polysaccharide substrates, followed by High Performance Anion Exchange with Pulsed Amperometric Detection (HPAEC-PAD) pattern analysis of the complex mixture of released products. This complexity is due to the presence of both neutral and oxidized sugars and the variation in their degree of polymerization (DP). On-column decomposition of the C4-oxidized sugars can make it extra challenging [24].

Here, we present an indicator diagram to quantify the impact of a rational engineering strategy on regioselectivity. The diagram was successfully used to confirm the role of *Hj*LPMO9A’s aromatic surface residues in the C1/C4 oxidation by site-directed mutagenesis.

## Materials and methods

### Genes and vectors

The genes for *Neurospora crassa* LPMO9C (Uniprot ID Q7SHI8) and *Phanerochaete chrysosporium* LPMO9D (H1AE14) were synthesized using the GeneArt gene synthesis service and codon optimized for *Pichia pastoris*. *P*. *pastoris* strain CBS7435 and the pPpT4 plasmid were provided by the Institute of Molecular Biotechnology in Graz, Austria. See S1 Table for further information.

### Construction of LPMO expression vectors

In all cases, the LPMO genes were codon optimized for *P*. *pastoris* and integrated into the pPpT4 vector downstream of the methanol-inducible AOX1 promotor, preceded by their native secretion signal and with a His_6_ tag attached to their C-terminus. The cloning of *Hypocrea jecorina LPMO9A* gene (O14405) and *PcLPMO9D* gene was described earlier [25]. The *NcLPMO9C* gene was inserted into the pPpT4 backbone using the CLIVA method [26]. All primers used are summarized in Table 1. All PCR fragments were constructed using *PfuUltra* High-Fidelity DNA Polymerase (Agilent). *Escherichia coli* strain BL21 (DE3) was used for plasmid construction and was transformed by electroporation.

**Table 1:** Primers used for cloning NcLPMO9C into pPpT4 expression vector.

Fragment	Primer	Sequence (5’ → 3’)
pPpT4_BB	pPpT4_BB_fwd	CATCAC*CATCAC*CATCACTAGGCGGCCGCTCAAGAG
	pPpT4_BB_rev	CGTTTC*GGAATT*CTTTCAATAATTAG
*NcLPMO9C*_gene	NcLPMO9C_fwd	AATTCC*GAAACG*ATGAAGACTGGTTCCATCTTGGCTGCTTTGGTTG
	NcLPMO9C_rev	GTGATG*GTGATG*TGGCAAACACTGGGAGTACCAGTC

BB = backbone; fwd = forward primer; rev = reverse primer;

* = phosphorothioate modification

After confirming the sequence (LGC Genomics), plasmid DNA was *Swa*I-linearized and transformed into freshly prepared electro-competent *P*. *pastoris* CBS7435 cells [27]. A *P*. *pastoris* control strain was prepared by transforming an empty *Swa*I-linearized pPpT4 vector into the wildtype strain. Transformants were grown on YPD plates supplemented with 100mg/L zeocin.

### Site-directed mutagenesis of *Hj*LPMO9A

Enzyme variants were obtained by site-directed mutagenesis of the *HjLPMO9A* sequence using a two-stage PCR reaction based on the Sanchis method using Q5 High-Fidelity DNA Polymerase (Bioke) [28]. Together with the variable mutagenic primers, the rev1 primer was used for the Y24A, F43A and W84A mutation, the rev2 primer for the Y211A mutation (primers in Table 2). After digestion of methylated template DNA with *Dpn*I, the mutagenized plasmid was transformed in *E*. *coli* BL21 (DE3) cells by electroporation.

**Table 2:** Primers used for the creation of mutants of the C1/C4 oxidizing LPMO *Hj*LPMO9A.

Primer	Sequence (5’ → 3’)
Y24A_fwd	CAACTACTTTCCCAGCTGAATCCAACCCACCAATC
F43A_fwd	TTGGACAACGGAGCTGTTTCTCCAGACGCTTACC
W84A_fwd	TGGGTTCCAGTTCCAGCTCCACATCCAGGTCCTATC
Y211A_fwd	GGTGTTTTGATCAACATCGCTACTTCCCCATTGAAC
rev1	ACCACCAGCAGGAGCAGAAG
rev2	GAAGAGGAGTGGGAAATACC

The codon subjected to mutagenesis is underlined. fwd = forward primer; rev = reverse primer.

After confirming the sequence (LGC Genomics), plasmid DNA was *Swa*I-linearized and transformed into freshly prepared electro-competent *P*. *pastoris* CBS7435 cells. Transformants were grown on YPD plates supplemented with 100 mg/L zeocin.

### Media

*E*. *coli* LPMO clones were cultivated in Luria-Bertani broth supplemented with 25 mg/L zeocin.

*P*. *pastoris* LPMO transformants were grown on BMGY medium (10 g/L yeast extract, 20 g/L peptone, 13.4 g/L yeast nitrogen base without amino acids, 100 mM potassium phosphate buffer pH6 and 10 g/L glycerol) and induced with BMM2Y (BMGY medium without glycerol but with 2% (v/v) methanol) and BMM10Y (with 10% (v/v) methanol).

*P*. *pastoris* strains were maintained on YPD plates (10 g/L yeast extract, 20 g/L peptone and 20 g/L glucose), if necessary supplemented with 100 mg/L zeocin.

### Deep-well plate screening for high-producing LPMO transformants

LPMO transformants were cultivated and expressed in 96-deep-well plates (Enzyscreen) tilted under an angle of 25° following the microscale protocol described by Jacobs et al. [29], with small modifications. Clones were grown in 250 μL BMGY for 60h at 28°C and 300 rpm to reach the stationary growth phase. Induction was started by adding 250 μL BMM2Y medium and maintained by spiking the cultures twice a day with 50 μL BMM10Y medium. After 5 days of induction, cultures were harvested by centrifugation at 1500 x g for 20 minutes (4°C). Extracellular expression was analyzed by SDS-PAGE of the culture supernatant.

### Cultivation and enzyme purification

The best-producing transformant was cultivated and expressed in a 250mL shake flask at 30°C and 200 rpm. Growth was started in 25 mL BMGY medium, induction followed 60 hours later by adding 25 mL BMM2Y medium. Methanol induction was maintained by adding two shots of 1 mL BMM10Y a day. After 5 days of induction cultures were harvested by centrifugation at 1500 x g for 20 minutes (4°C).

The culture supernatant was concentrated and washed with 10 mM sodium acetate buffer (pH 5) by ultrafiltration using 10 kDa Ultracel ultrafiltration disks (Millipore) in an Amicon stirred ultrafiltration cell. A final volume of 2 mL concentrated enzyme was obtained.

For protein purification, culture supernatant was first ultrafiltrated as described above, using PBS buffer (50 mM sodium phosphate and 300 mM sodium chloride at pH 7.4), to a final volume of about 5 mL. After adding imidazole to a concentration of 10 mM, the ultrafiltrated samples were applied to 1.5 mL equilibrated Ni-NTA agarose slurry (MC-lab) in 10 mL purification columns. The columns were incubated for 1 h at 4°C while gently rotating to allow binding to the resin. Next, the columns were washed with 3 x 8 mL of 20 mM imidazole in PBS-buffer and the protein was finally eluted in 10 mL of 250 mM imidazole solution. The buffer was exchanged for 10 mM sodium acetate (pH 5) and the sample was concentrated to 2 mL using Vivaspin 20 columns with 10 K PES membrane (Sartorius).

For large scale production of wildtype *Hj*LPMO9A, a bioreactor cultivation was performed in a 5L fermentor vessel (Infors HT Labfors) starting with 4L of BSM medium containing 6% glycerol at 30°C, 2 vvm of air and 1200rpm. After the batch phase, six methanol pulses between 0.5–2.0% were performed for induction (following procedure of Dietzsch et al.[30]). After methanol induction, the protein was obtained by centrifuging the fermentation broth for 15 min at 5000 rpm. The resulting supernatant was concentrated by diafiltration (PALL Centramate Omega, 0.1 m^2^, 10 kDa cut-off) using 20 mM PBS buffer (500 mM NaCl) supplemented with 20 mM imidazole and further filtrated using 0.2 μm filters (Roth). The concentrated culture supernatant was purified by immobilized metal affinity chromatography (IMAC) using 3x 1 mL HisTrap columns (GE). After elution using 20 mM PBS buffer (500 mM NaCl) with 500 mM imidazole, the buffer was exchanged for 10 mM sodium acetate buffer (pH 5) using Amicon-Ultra 15 spin tubes with 10 kDa cut-off (Millipore). Although this protocol had a higher yield, it did not have additional benefits in removing the endoglucanase background and was therefore not routinely pursued.

### Protein analysis and concentration

Culture supernatant and concentrated enzyme were analyzed using 12% SDS-PAGE gels to confirm the presence of the LPMO enzyme. Protein bands were visualized by staining with colloidal Coomassie G-250 stain (Bio-Rad) and the PageRuler prestained protein ladder (Thermo Fisher Scientific) was used for mass determination.

A dilution series of bovine serum albumin (0.3–0.05 mg/L) was added to each gel to get an estimation of the protein concentration of the ultrafiltrated culture supernatant, using the digital imaging software ImageJ as described earlier [25,31].

The concentration of purified enzyme was measured using a Nanodrop device with extinction coefficients of 54360 M^-1^.cm^-1^, 52870 M^-1^.cm^-1^ and 52870 M^-1^.cm^-1^ for *Hj*LPMO9A wildtype, variant Y24A and variant Y211A, respectively.

### Activity tests and product analysis

Reaction mixtures of 500 μL contained 0.5% Phosphoric Acid Swollen Cellulose (PASC) and 1 mM ascorbic acid in 1 mM sodium acetate (*Hj*LPMO9A and *Nc*LPMO9C) or 1 mM MES buffer (*Pc*LPMO9D) at a final pH of 4.5 and 6.1, respectively. The regioselectivity indicator diagram was developed by using a *Hj*LPMO9A dilution series of 20–250 μg enzyme. All measurements and calculations for the development of the indicator diagram were done independently in triplicate. In the final indicator diagram the slope of the WT line is calculated as the average of the independently calculated lines.

The activity tests with *Pc*LPMO9D and *Nc*LPMO9C were carried out using 12.5–125 μg and 15–50 μg respectively. Mutant assays were performed using 65, 32 and 12.5 μL of concentrated culture supernatant per 500 μL, corresponding to an enzyme load between 20–200 μg. Reactions with purified *Hj*LPMO9A and its variants Y24A and Y211A were performed using 20–200 μg LPMO. Reactions were incubated for 4 hours (9 h for the purified enzymes) at 50°C (*Hj*LPMO9A and *Pc*LPMO9D) or 40°C (*Nc*LPMO9C) while shaking at 1400 rpm using an Eppendorf Thermomixer.

25 μL samples were taken every 30 min and the reaction was stopped by heat-inactivation (10 min at 95°C), which was proven to inactivate the enzymes. Samples were diluted 10-fold before HPAEC-PAD analysis using the protocol described by Forsberg et al., using a CarboPac PA-10 column (Dionex, Sunnyvale, CA, USA) instead of the CarboPac PA1 column used in the original protocol [32].

## Results and discussion

### Expression of 3 fungal LPMOs in *P*. *pastoris* and their regioselectivity

A characterized member of each LPMO regioselectivity type was recombinantly expressed in *P*. *pastoris*, namely *P*. *chrysosporium* LPMO9D [33], *N*. *crassa* LPMO9C [19,34] and *T*. *reesei* LPMO9A [25] as C1, C4 and C1/C4-oxidizer, respectively. All LPMOs were detected at a molecular mass higher than their calculated masses, most likely due to glycosylation (S1 Fig).

**Fig 2:**
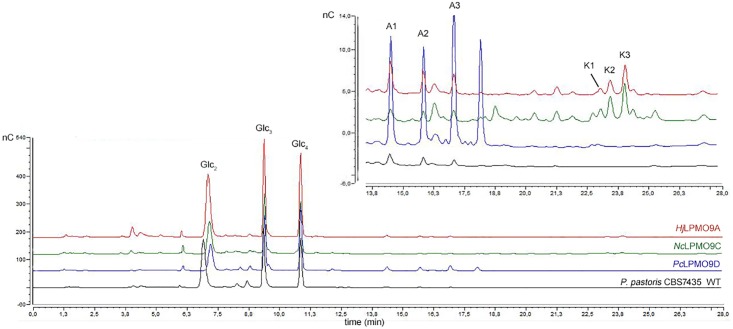
HPAEC-PAD chromatograms of all 3 LPMO representatives. Activity of *Hj*LPMO9A (red), *Nc*LPMO9C (green), *Pc*LPMO9D (blue) and wildtype *P*. *pastoris* CBS7435 broth (black) on 0.5% PASC in the presence of 1 mM ascorbic acid as reducing agent. The six signals (A1-3 and K1-3) that will be evaluated for their use as indicator signal are indicated in the top chromatogram.

The soluble reaction products released upon incubation of the three LPMOs with the polymeric substrate PASC were analyzed by HPAEC-PAD (Fig 2). HPAEC-PAD is an excellent method for the detection of C1-oxidized sugars, however, for C4-oxidized sugars, the detection is limited. Indeed, the latter require a higher gradient of sodium acetate for their elution, resulting in a lower pH and weaker response on the gold electrode [35]. Next to this, the detection of C4-oxidized oligosaccharides is further hampered by tautomerization and chemical modification under alkaline conditions [3]. An alternative to HPAEC is porous graphitized carbon (PCG) chromatography, which gives excellent separation of C1- and C4-oxidized cello-oligosaccharides, but requires mass spectrometry (MS) detection for the separation of native and C4-oxidized products. For this reason it is less interesting as screening tool, but nevertheless a very promising method in LPMO regioselectivity research [24].

The HPAEC-PAD chromatograms confirm the regioselectivity of *Pc*LPMO9D as strict C1-oxidizer and *Hj*LPMO9A as C1/C4-oxidizer, but signals of C1-oxidized products are also observed for the strict C4-oxidizer *Nc*LPMO9C. However, this C1-oxidation response is not significantly higher than the C1-oxidative background activity in the *P*. *pastoris* CBS7435 wildtype strain (see S1 File). Furthermore, the high amounts of cellobiose, cellotriose and cellotetraose released from PASC (visible as the 3 large peaks in Fig 2), point towards the presence of *P*. *pastoris* endoglucanase activity in the ultrafiltrated culture supernatants used in our analyses. This hydrolytic activity results in faster release of native as well as oxidized oligosaccharides, and enables short incubation times for screening, as will be demonstrated further on.

### Development of quantitative regioselectivity indicator diagram

The underlying causes for differences in regioselectivity in LPMOs remain unclear, although several hypotheses have been formulated in literature. The copper binding site is reported to play a role, as does the positioning of aromatic residues and N-glycans in the planar surface [17,18,20]. Based on multiple sequence alignments and phylogenetic studies, three regioselectivity classes have previously been proposed [19,36], but several LPMOs have meanwhile been found to deviate from these predictions [5,19,37]. Bennati-Granier et al. have, therefore, suggested to limit the alignments to residues that interact directly with the substrate [5]. Mutagenesis and structural studies using soluble substrates like those performed by Frandsen et al. [12] together with further biochemical characterization of LPMOs should make it possible to unravel the secrets to C1/C4-oxidation ratio in LPMOs.

Site-directed mutagenesis enables the investigation of structure-function relationship in enzymes, but will, in the context of regioselectivity, depend highly on an enzyme assay that can unambiguously demonstrate a shift in product pattern. To that end, a quantitative relationship needs to be established between the formation of both product types.

**Fig 3:**
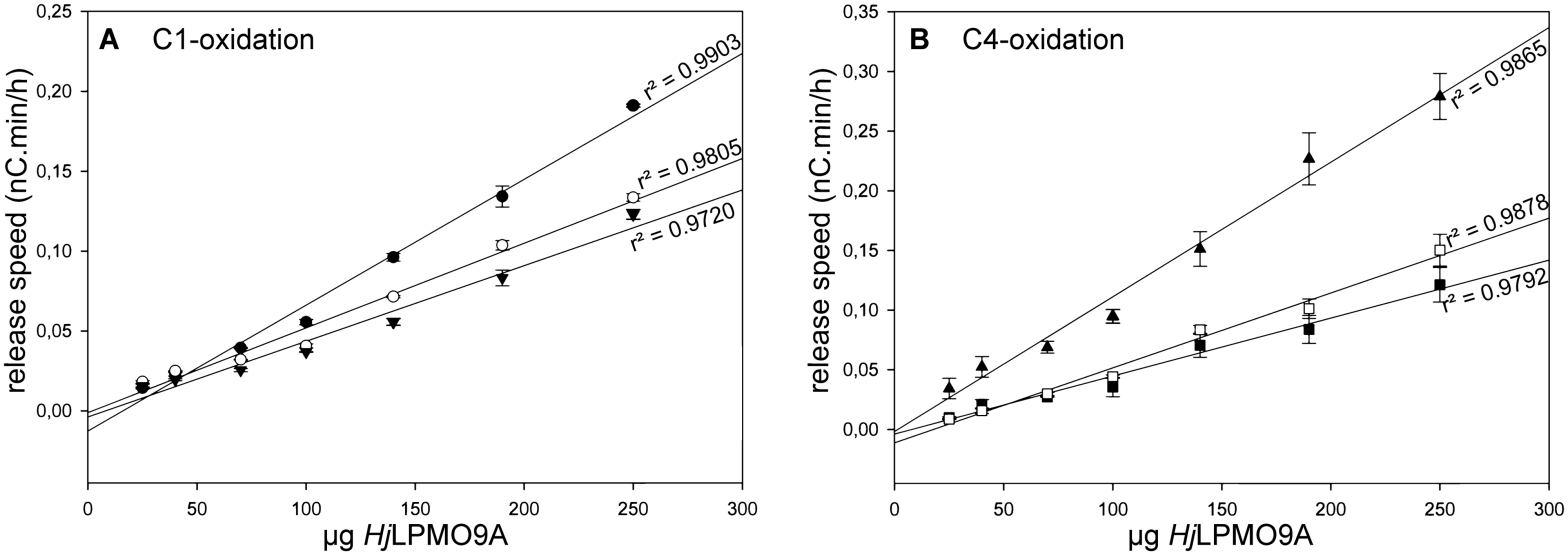
Correlation between release speeds of aldonic acid / 4-ketoaldose peaks and the *Hj*LPMO9A LPMO load in 500 μL reaction mixture. Graph A represents the aldonic acid peaks (● = A1, ○ = A2, ▼ = A3); Graph B represents the 4-ketoaldose peaks (■ = K1, □ = K2, ▲ = K3).

Using a dilution series of *Hj*LPMO9A, the formation of aldonic acids and 4-ketoaldoses was monitored during a 4 hour timespan (see Fig 2 for selected peaks, and S2 Fig for the time courses of all six). The rate of formation was calculated for each product and was found to correlate linearly with the enzyme load (Fig 3). It is noteworthy that not only the aldonic acids, for which HPAEC-PAD analysis is well established, but also the 4-ketoaldose peaks are proportional to the enzyme concentration. Absolute quantification will require authenticated standards of C4-oxidized cello-oligosaccharides, which were recently generated for the first time using semi-preparative porous graphitized carbon chromatography (PGC) [24]. Muller et al. developed another method for quantifying Glc4GemGlc (C4-oxidized cellobiose) using a diagnostic HPAEC signal [38], and although our experiments do not offer absolute quantification of the C4-oxidized products, the linear correlation with the enzyme concentration does imply applicability of the selected peaks for regioselectivity mutagenesis studies.

Next, the correlation between the aldonic acid and the 4-ketoaldose reaction rates was determined and confirmed to be linear for all product combinations. However, as working with fungal LPMOs is already quite time-consuming and labor-intensive, analysis of mutant enzymes should be made as fast and simple as possible. Therefore, we aimed at developing an indicator diagram with just one signal for C1- and one for C4-oxidized sugars.

After extensively examining the HPAEC-PAD chromatograms obtained with the different LPMO representatives, it became clear that both the A1 and A3 peak suffer from very small partially overlapping neighboring peaks. Re-integration of these A-peaks did result in good correlation between concentration and release speed as demonstrated above. This does, however, result in extra data processing time, which is undesirable in future mutagenesis experiments. The A2 signal, in contrast, does not suffer from overlapping peaks or from background noise, making it the best aldonic acid indicator. In turn, the K1 and K2 peaks overlap partially, while the K3 peak is the highest and always the first to pass the detection threshold. Therefore, the latter will be selected as second indicator signal.

**Fig 4:**
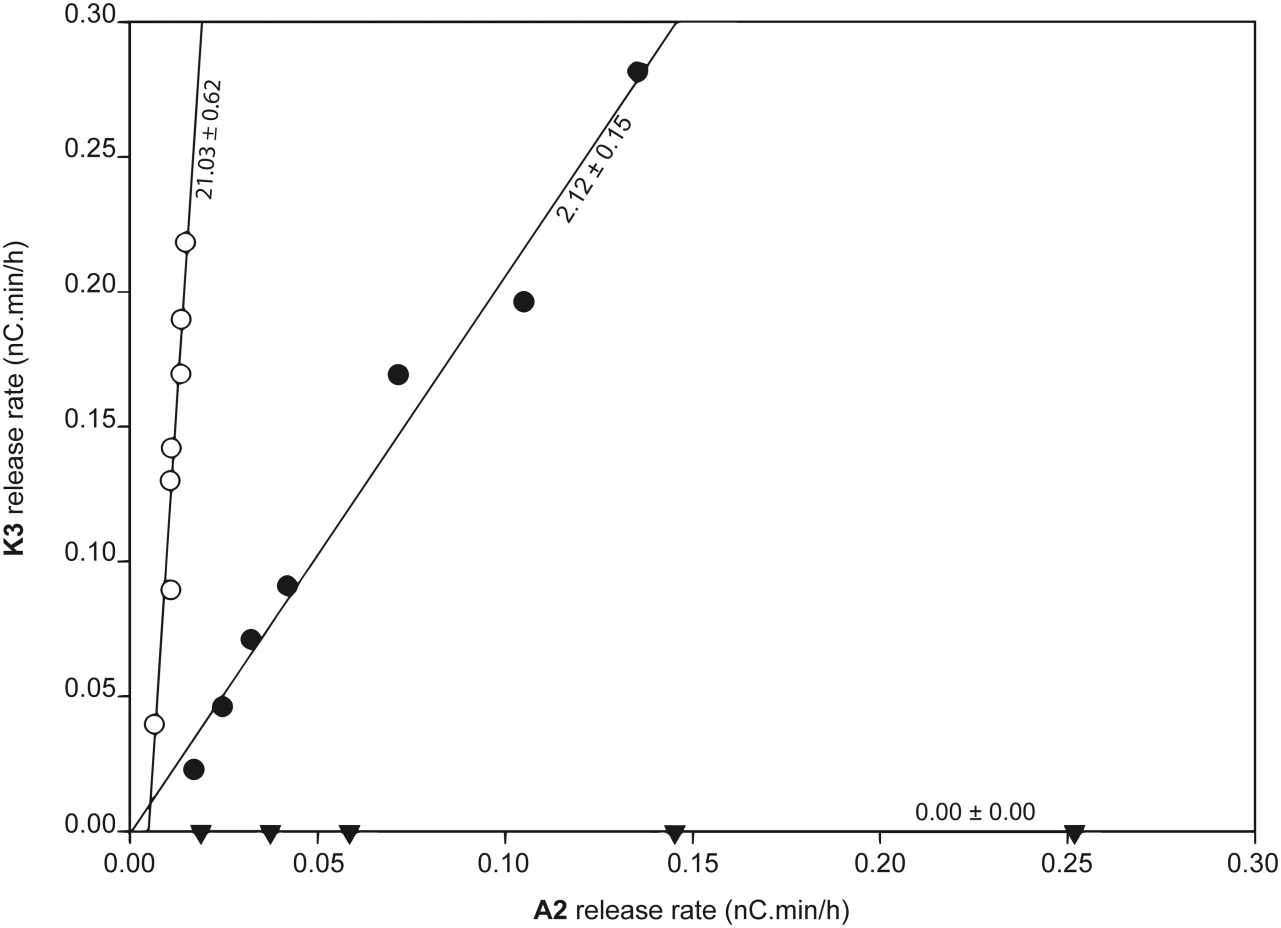
Indicator diagram applied to the three LPMO regioselectivity types. Preliminary evaluation of the indicator diagram was done by incubating a member of each LPMO regioselectivity type on PASC: ● = *Hj*LPMO9A (C1/C4-oxidizer, 1.2–12 μM), ▼ = *Pc*LPMO9D (C1-oxidizer, 1–10 μM), ○ = *Nc*LPMO9C (C4-oxidizer, 0.9–2.8 μM).

An indicator diagram was then established using the values of the A2 and K3 release rates as ordinate and abscissa, respectively (Fig 4). The slope of the regression line is considered to be a measure of the C4/C1-oxidation ratio.

Preliminary validation of the proposed regioselectivity indicator diagram was done using the C1-oxidizing *Pc*LPMO9D and C4-oxidizing *Nc*LPMO9C enzyme. As no 4-ketoaldose peaks were detected, all *Pc*LPMO9D measurements lie on the X-axis, confirming its C1-oxidizing activity. As a result of C1-oxidative background activity in wildtype *P*. *pastoris* CBS7435, *Nc*LPMO9C measurements do not line up with the Y-axis. However, this does not hamper the screening of variants for relative changes in product profile and only needs to be taken into account when determining the absolute regioselectivity of the wildtype enzyme (see S1 File for more details).

### Aromatic surface residues important for regioselectivity in *Hj*LPMO9A

Aromatic surface residues are known to play an important role in carbohydrate-binding structures of cellulases and chitinases [39]. In CBP21, the single point mutation Y54A resulted in a lowered affinity for β-chitin, expanding the relevance of aromatic surface exposed residues to LPMOs [11]. Such residues are not only believed to play a role in binding of the substrate, but also in its exact positioning. Based on the structure of an LPMO in complex with an oligosaccharide ligand, it was suggested that very small shifts in substrate orientation could have a profound influence on the C1/C4-oxidation balance [12]. Using various bio-informatics approaches, Moses et al. independently suggested that the composition of surface aromatic residues determines LPMO regioselectivity, with C1-oxidizing LPMOs having the highest surface aromaticity [21].

**Fig 5:**
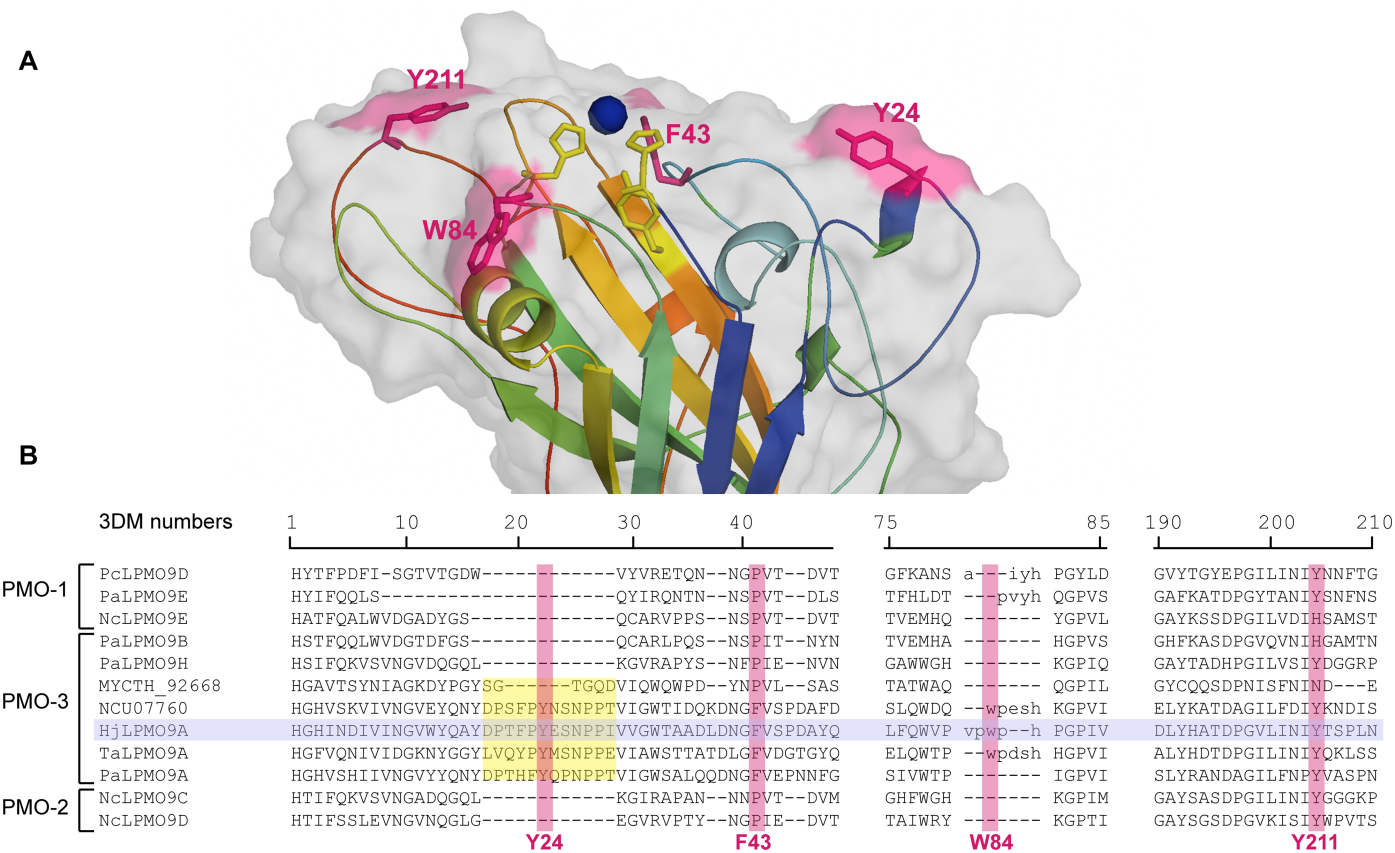
Aromatic surface residues in the C1/C4-oxidizing *Hj*LPMO9A. (A) Homology model of *Hj*LPMO9A (based on 3ZUD as template) with the aromatic surface residues selected for alanine scanning in pink stick representation. Active site residues are shown as yellow sticks, the copper ion as a blue sphere. (B) 3DM structure based multiple sequence alignment [40] of AA9 characterized LPMOs with known regioselectivity. The residues aligned with the Y24, F43, W84 and Y211 aromatic surface residue of *Hj*LPMO9A are highlighted in pink. Residues in the 3DM core alignment are represented by capitals, the alignment of structurally variable regions are in lower case. The insertion typical for most C1/C4-oxidizing LPMOs is marked in yellow.

As no crystal structure is available, a homology model, based on 3ZUD as a template, was used to identify all surface exposed aromatic residues in *Hj*LPMO9A (Fig 5). Through site-directed mutagenesis, Y24, F43, W84 and Y211 were then substituted by alanine. For the evaluation of the resulting variants, the formation rates of the A2 and K3 peaks were determined at three different enzyme concentrations. In all cases, the A2 and K3 rates demonstrate good correlation with each other, although the slopes of the indicator lines differ from that of the wildtype (Table 3). In particular, Y24A and Y211A prove to have a significant effect on the regioselectivity of *Hj*LPMO9A, while F43A has only a minor effect and W84A no effect at all. Interestingly, the corresponding positions were already suggested to play a role in substrate binding in other LPMOs. Y24 is part of a helix that is often present in C1/C4-oxidizers, but never in enzymes with single oxidative capacity. By removing this helix, NCU07760 loses most of its C4-oxidative capacity, although the mutation Y24G by itself did not alter regioselectivity [19]. In *Nc*LPMO9C, Y204 (Y211 in *Hj*LPMO9A), located on the LC-loop, was demonstrated to be important for interactions near subsite -3/-4, but due to the lack of mutagenesis studies, residues important for C4-oxidation have not been identified yet [10].

**Table 3:** Effect of mutating aromatic surface residues on *Hj*LPMO9A regioselectivity. This effect is determined by comparing the C1/C4-oxidation ratio (slope in the indicator diagram) of the wildtype enzyme (1.4–14 μM) and the variants (1.2–12 μM).

*Hj*LPMO9A variant	Slope in indicator diagram	Effect
WT	2.12 ± 0.15	
Y24A	1.30 ± 0.11	More C1-oxidation
F43A	1.60 ± 0.16	More C1-oxidation
W84A	1.99 ± 0.18	None
Y211A	4.83 ± 0.14	More C4-oxidation

**Fig 6:**
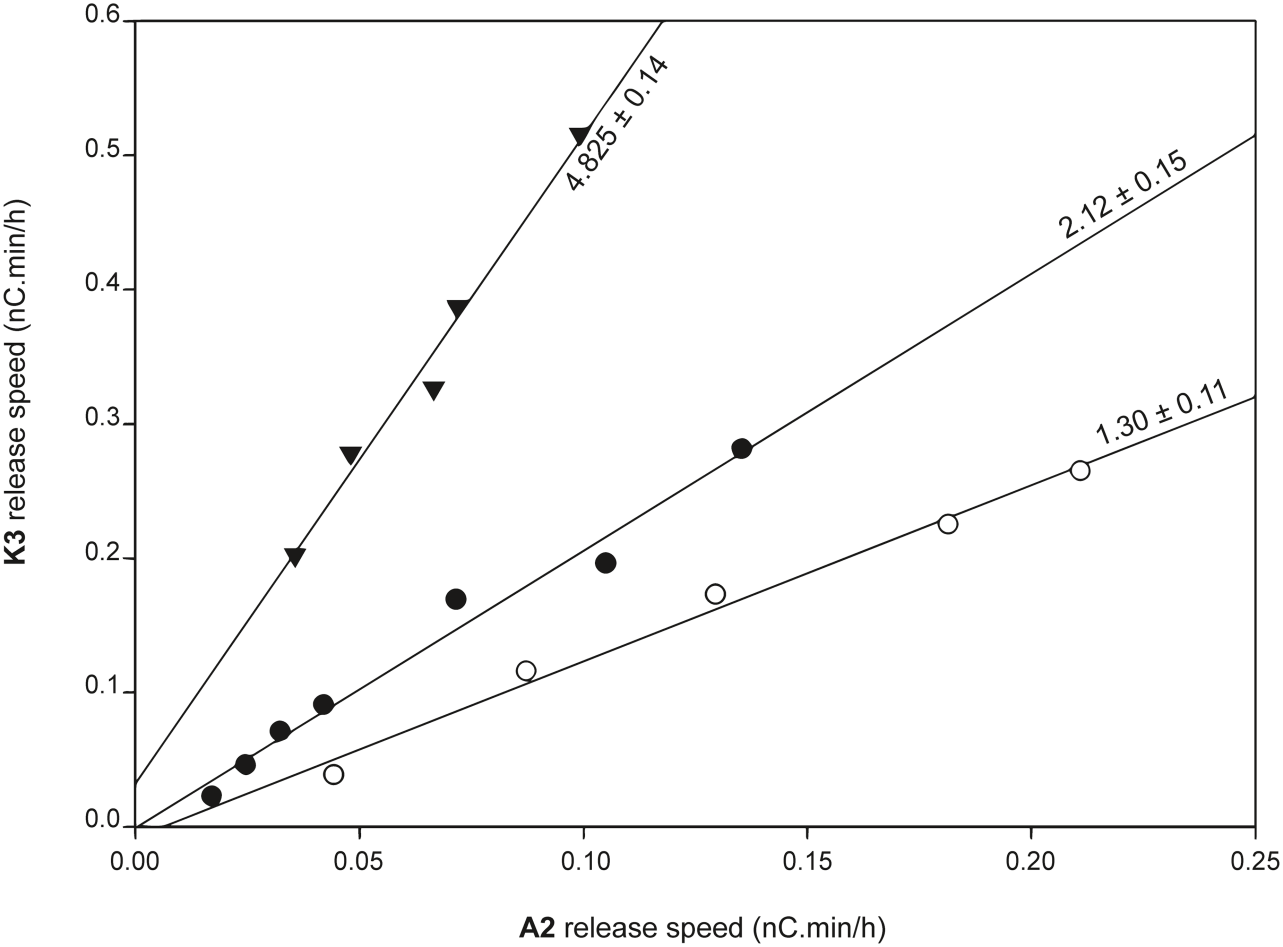
Indicator diagram demonstrates role of aromatic residues in LPMO regioselectivity. Slopes, which are a measure of the ratio of C4/C1-oxidation, are listed next to the regression line, together with their standard deviation. ● = *Hj*LPMO9A wildtype (1.4–14 μM), ○ = Y24A variant (1.2–12 μM), ▼ = Y211 (1.2–12 μM) variant.

For the two mutations with the highest effect on regioselectivity, three more enzyme concentrations were examined and included in the indicator diagram (Fig 6). This confirmed that by mutating Y24, the enzyme’s C1-oxidative capacity is increased, while the C4-oxidative capacity does not seem to be affected. Vice versa, mutation Y211A results in higher release rates of C4-oxidation products (even higher than ever observed with the wildtype enzyme), accompanied by a slight decrease of C1-oxidation rate. It thus appears that Y24 and Y211 are balancing out the C4/C1-oxidation ratio: by losing one, the effect of the other becomes more prominent.

In order to get an idea of the overall activity of the *Hj*LPMO9A enzyme and its variants, the total amount of oxidized species was compared based on their peak areas in HPAEC-PAD chromatograms. The results are shown in S3 Fig, confirming that our previous observed regioselectivity effects can be broadened to all oxidized signals. The results also show that the mutations do not negatively affect the overall activity.

To further validate our findings, the effect on regioselectivity was also determined with purified enzymes, in which the endoglucanase background was no longer present. In that case, the HPAEC-PAD chromatograms became more complex, and showed a clear shift towards products with higher DP (see S4 and S5 Figs for comparison). Nevertheless, the new indicator diagram, based on A1-3* and K1-3* as diagnostic signals, confirmed the effect on C1/C4-oxidative selectivity for both mutations (see S6 Fig).

The results obtained with variant Y24A are in agreement with the effect observed after deleting the characteristic helix in enzyme NCU07760, although the single mutation Y24G did by itself not alter regioselectivity [19]. Most likely, the influence of this residue is less pronounced in the framework of NCU07760, and hence more difficult to discern.

### Applicability of the regioselectivity indicator diagram

Since their discovery in 2010, many studies have addressed the structure and mechanism of LPMOs, but very few publications describe enzyme engineering experiments. One of the reasons could be the lack of a convenient screening assay, meaning that such studies would have to rely on lengthy incubations and exhaustive analyses of HPAEC-PAD chromatograms. The indicator diagram established here could partially alleviate this burden, as the incubation time can be kept relatively short (4 hours) and the interpretation is based on just 2 signals in the chromatogram. In future experiments, the workload could be reduced even more by using only one enzyme concentration instead of the complete indicator line that has been examined here to validate the diagram. This experimental cut down would indeed be justified as the relationship between the A2- and K3-rates was found to be constant over a wide range of enzyme concentrations (Fig 6).

## Conclusions

Expressing a representative member of each LPMO class in *P*. *pastoris* and time-course monitoring of cellulose cleavage led to the establishment of an indicator diagram that enabled quantitative determination of their regioselectivity. As proof of concept, the effect of four point mutations was examined in detail by performing activity tests at different enzyme concentrations. By using this diagram, we were able to demonstrate that aromatic surface residues are important for regioselectivity in *Hypocrea jecorina* LPMO9A. This is in accordance with the hypothesis that by carefully orienting the oxidative force of the copper ion towards the C1 or C4 glycosidic position, these aromatic residues can determine the exact site of oxidation [14]. Further mutagenesis experiments, targeting other positions and enzymes, should reveal more features and motifs specific for C1/C4-oxidation.

## Supporting information

S1 Fig SDS-PAGE analysis of the three LPMO regioselectivity representatives. Lane 1: *Hypocrea jecorina* LPMO9A native enzyme; lane 2: *Phanerochaete chrysosporium* LPMO9D native enzyme; lane 3: *Neurospora crassa* LPMO9C native enzyme; lane 4: PageRuler prestained protein ladder (Thermo Scientific); lane 5: histag purified *Hj*LPMO9A; lane 6: histag purified *Hj*LPMO9A variant Y24A; lane 7: *Hj*LPMO9A variant Y211A.
(DOCX)

S2 Fig Time course monitoring of aldonic acid and 4-ketoaldose peaks. Time courses of the three aldonic acid (A1, A2 and A3) and three 4-ketoaldose (K1, K2, K3) peaks released upon incubation of PASC with a dilution series of *Hj*LPMO9A.
(DOCX)

S3 Fig HPAEC-PAD analysis of oxidized cello-oligosaccharides released from PASC by *Hj*LPMO9A and its variants. The total amount of oxidized cello-oligosaccharides released by 70μg of LPMO after 2.5h incubation at 50°C is compared to gain insight in the overall activity changes of the mutations. The values are means of three replicates, error bars correspond to a cumulated total standard deviation (error bar = ± total Stdev, with total Stdev = √(Stdev_1_^2^ + Stdev_2_^2^ + Stdev3^2^)).
(DOCX)

S4 Fig HPAEC-PAD chromatograms (enzyme tests with culture supernatant). Chromatograms of wildtype *Hj*LPMO9A and regioselectivity mutants Y24A (with higher C1-oxidative capacity) and Y211A (with higher C4-oxidative capacity). Two time points (after 1h and 4h incubation) are shown for each enzyme variant.
(DOCX)

S5 Fig HPAEC-PAD chromatograms (enzyme tests with pure enzymes). HPAEC-PAD chromatograms of wildtype *Hj*LPMO9A and regioselectivity mutants Y24A (with higher C1-oxidative capacity) and Y211A (with higher C4-oxidative capacity) after histag purification. A control sample only containing PASC, 1mM ascorbic acid and buffer (without enzyme) was run to verify the enzyme preparations lost their endoglucanase background activity. Two time points (after 1h and 9h incubation) are shown for each enzyme variant.
(DOCX)

S6 Fig The indicator diagram for purified enzymes. This indicator diagram was obtained for wildtype *Hj*LPMO9A and mutants Y24A (with higher C1-oxidative capacity) and Y211A (with higher C4-oxidative capacity) after histag purification and confirms the effect on regioselectivity of the point mutations.
(DOCX)

S1 File Details on the C1-oxidative background activity in wildtype *P*. *pastoris* and its effect on the indicator diagram. (DOCX)

S1 Table DNA sequences. Sequence of *Neurospora crassa* LPMO9C and *Phanerochaete chrysosporium* LPMO9D.
(DOCX)
