# Supplementary material for: A quantitative indicator diagram for lytic polysaccharide monooxygenases reveals the role of aromatic surface residues in HjLPMO9A regioselectivity
Source: PLoS One. 2017 May 31;12(5):e0178446. doi: 10.1371/journal.pone.0178446 (PMC5451062; doi:10.1371/journal.pone.0178446)
Supplement: S1 Fig — Lane 1: Hypocrea jecorina LPMO9A native enzyme; lane 2: Phanerochaete chrysosporium LPMO9D native enzyme; lane 3: Neurospora crassa LPMO9C native enzyme; lane 4: PageRuler prestained protein ladder (Thermo Scientific); lane 5: histag purified HjLPMO9A; lane 6: histag purified HjLPMO9A variant Y24A; lane 7: HjLPMO9A variant Y211A. (DOCX) [file pone.0178446.s001.docx]

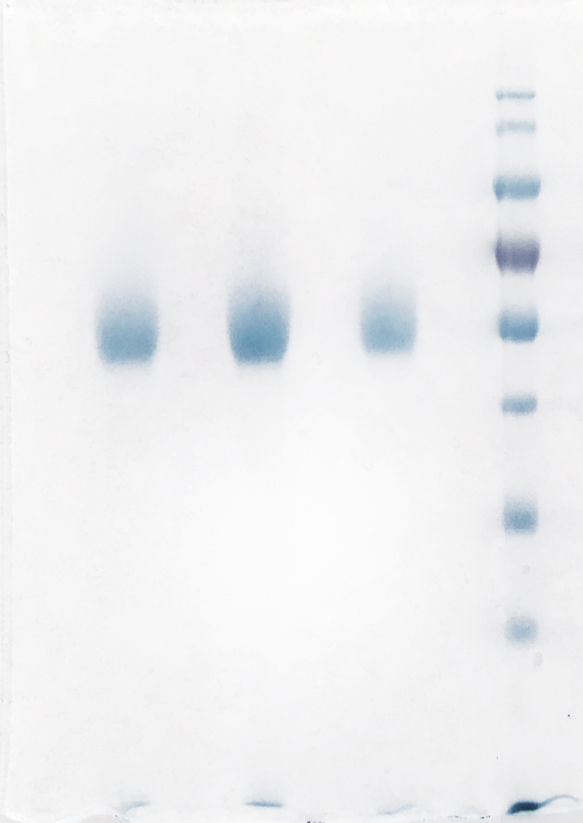

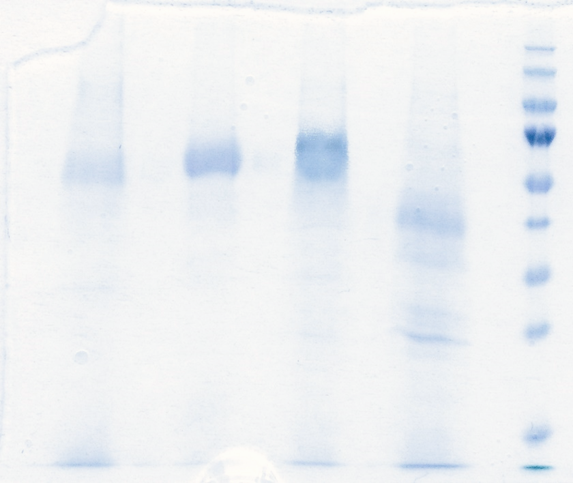


1 2 3 4 5 6 7

**S1 Figure. SDS-PAGE analysis of the three LPMO regioselectivity representatives**. Lane 1: *Hypocrea jecorina* LPMO9A native enzyme; lane 2: *Phanerochaete chrysosporium* LPMO9D native enzyme; lane 3: *Neurospora crassa* LPMO9C native enzyme; lane 4: PageRuler prestained protein ladder (Thermo Scientific); lane 5: histag purified *Hj*LPMO9A; lane 6: histag purified *Hj*LPMO9A variant Y24A; lane 7: *Hj*LPMO9A variant Y211A.
