# Supplementary material for: A quantitative indicator diagram for lytic polysaccharide monooxygenases reveals the role of aromatic surface residues in HjLPMO9A regioselectivity
Source: PLoS One. 2017 May 31;12(5):e0178446. doi: 10.1371/journal.pone.0178446 (PMC5451062; doi:10.1371/journal.pone.0178446)
Supplement: S2 Fig — Time courses of the three aldonic acid (A1, A2 and A3) and three 4-ketoaldose (K1, K2, K3) peaks released upon incubation of PASC with a dilution series of HjLPMO9A. (DOCX) [file pone.0178446.s002.docx]

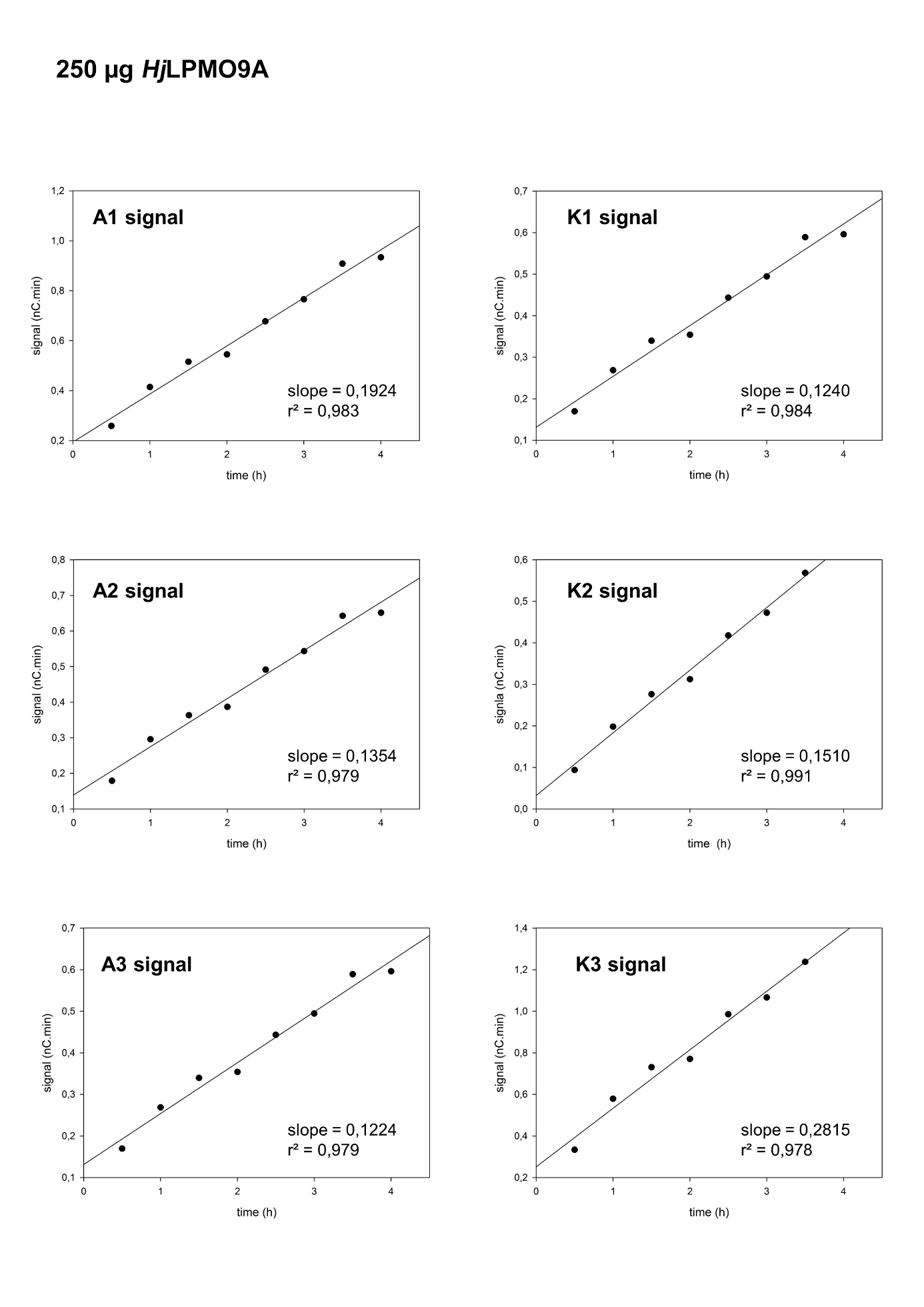


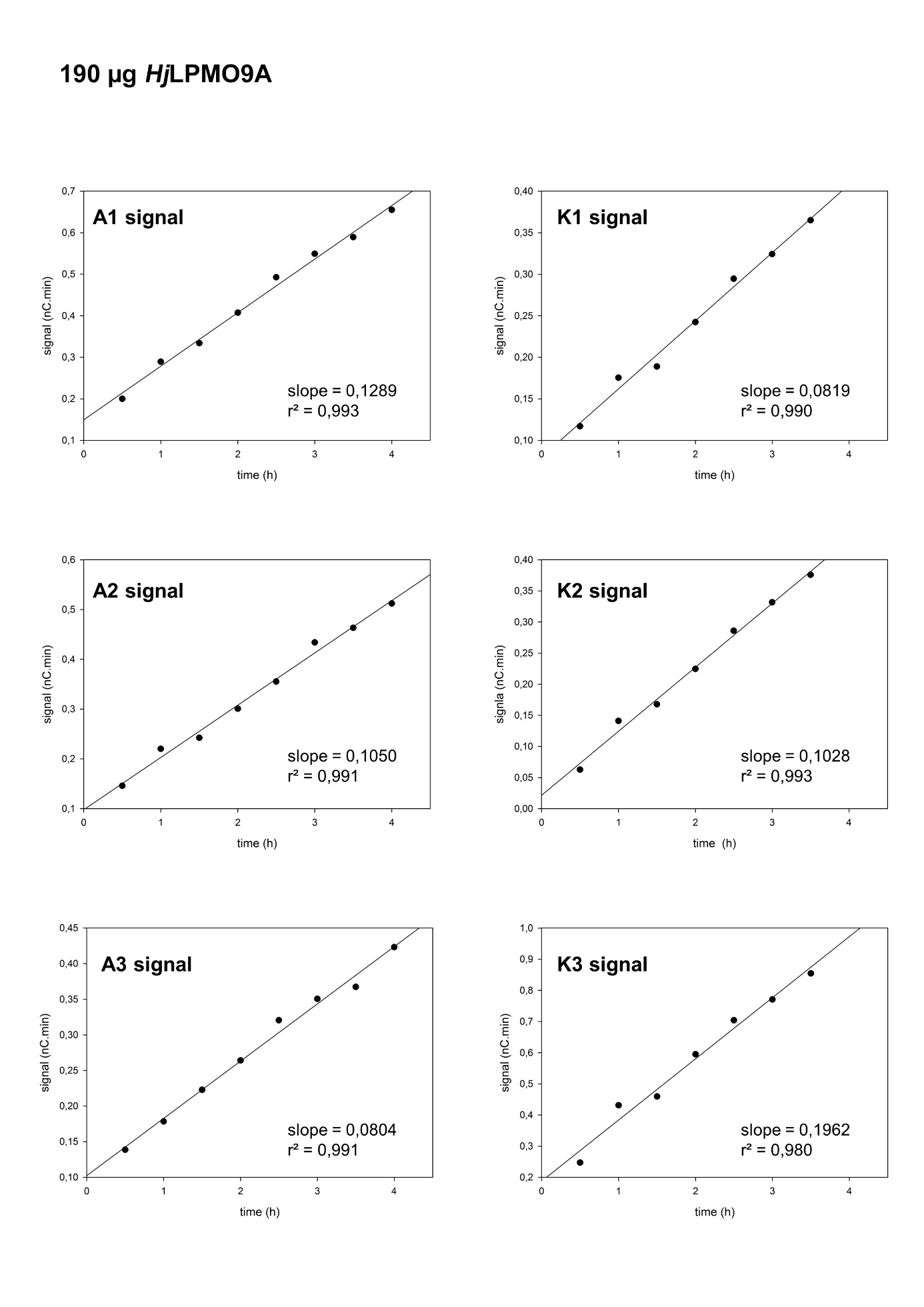


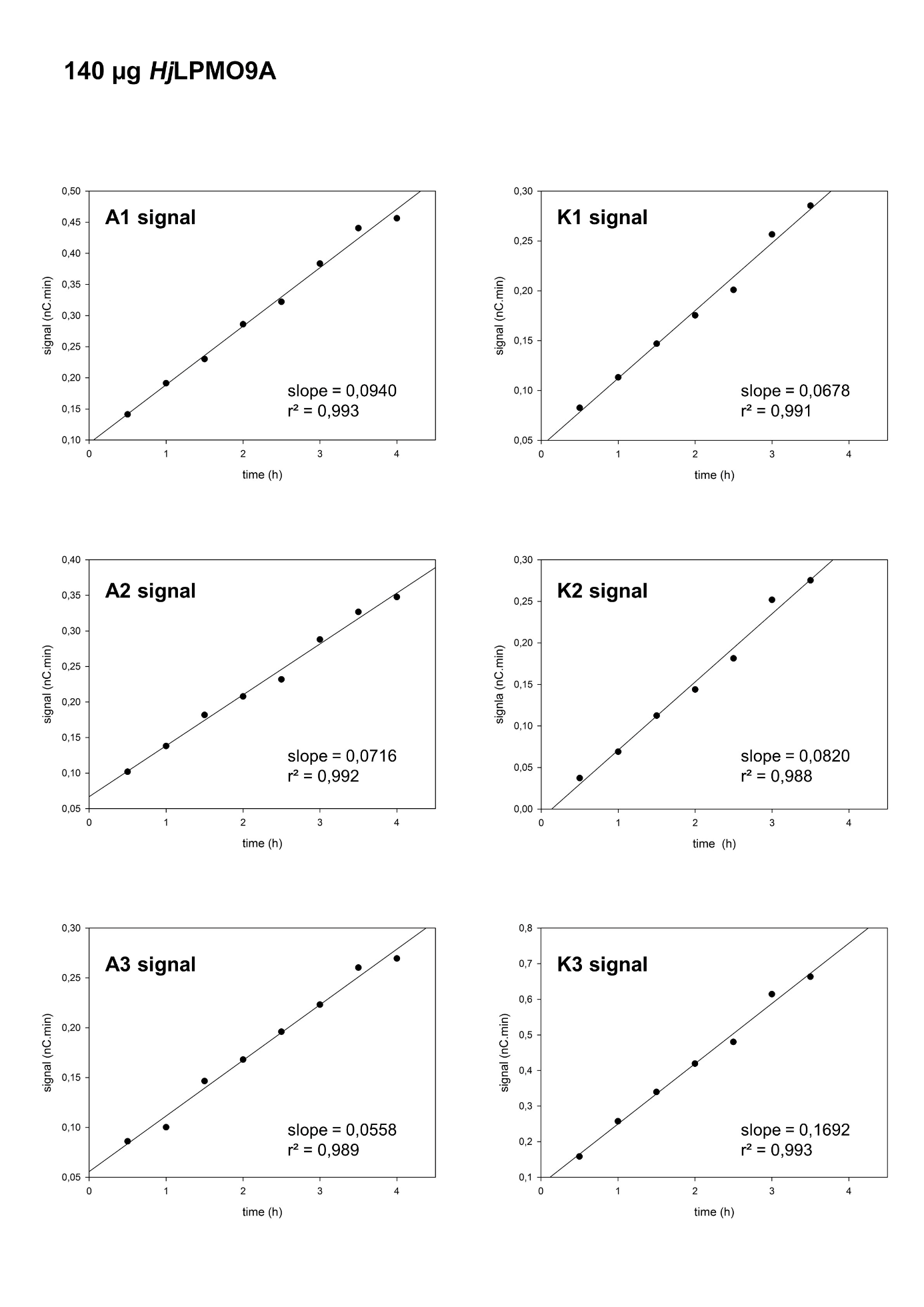


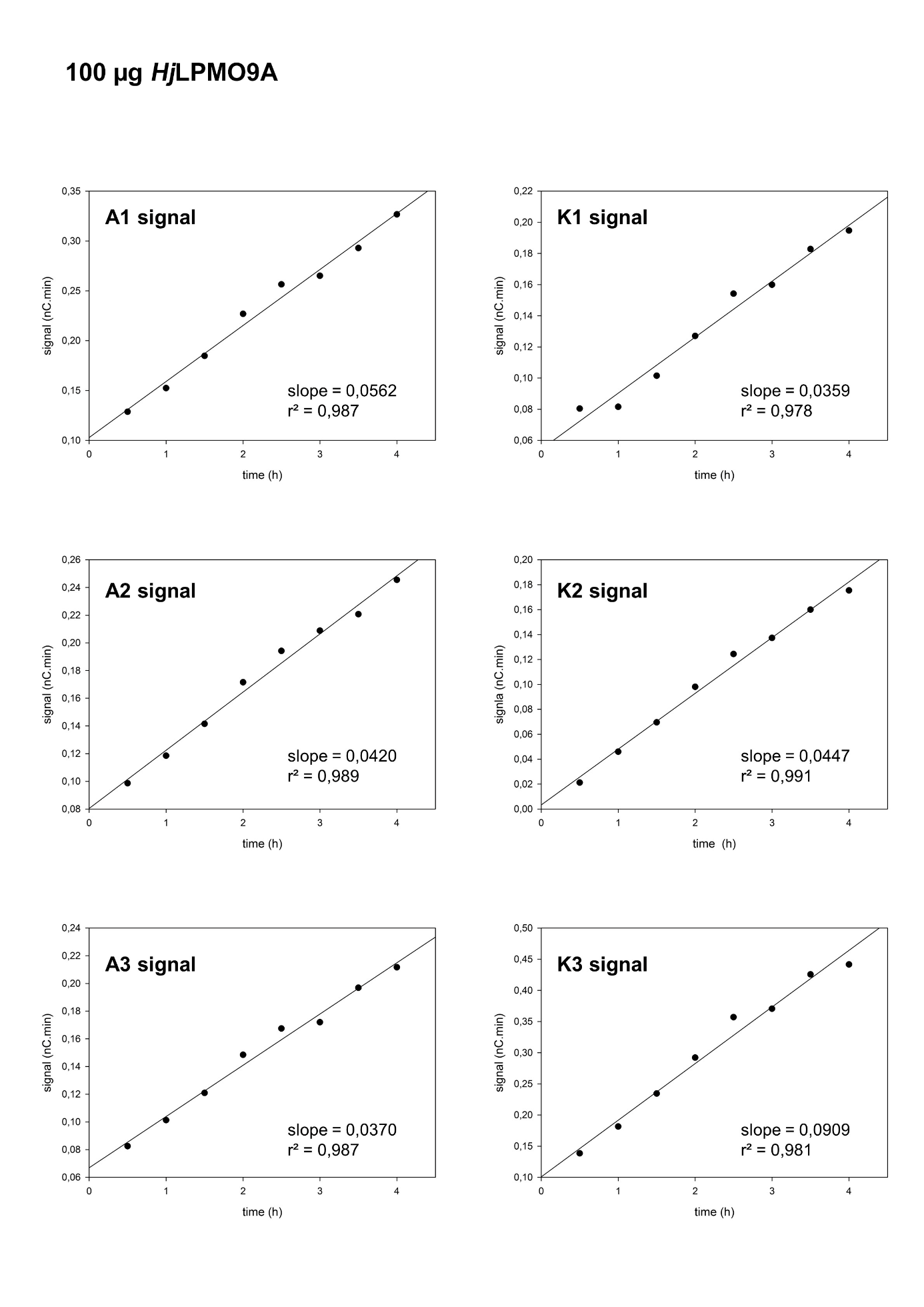


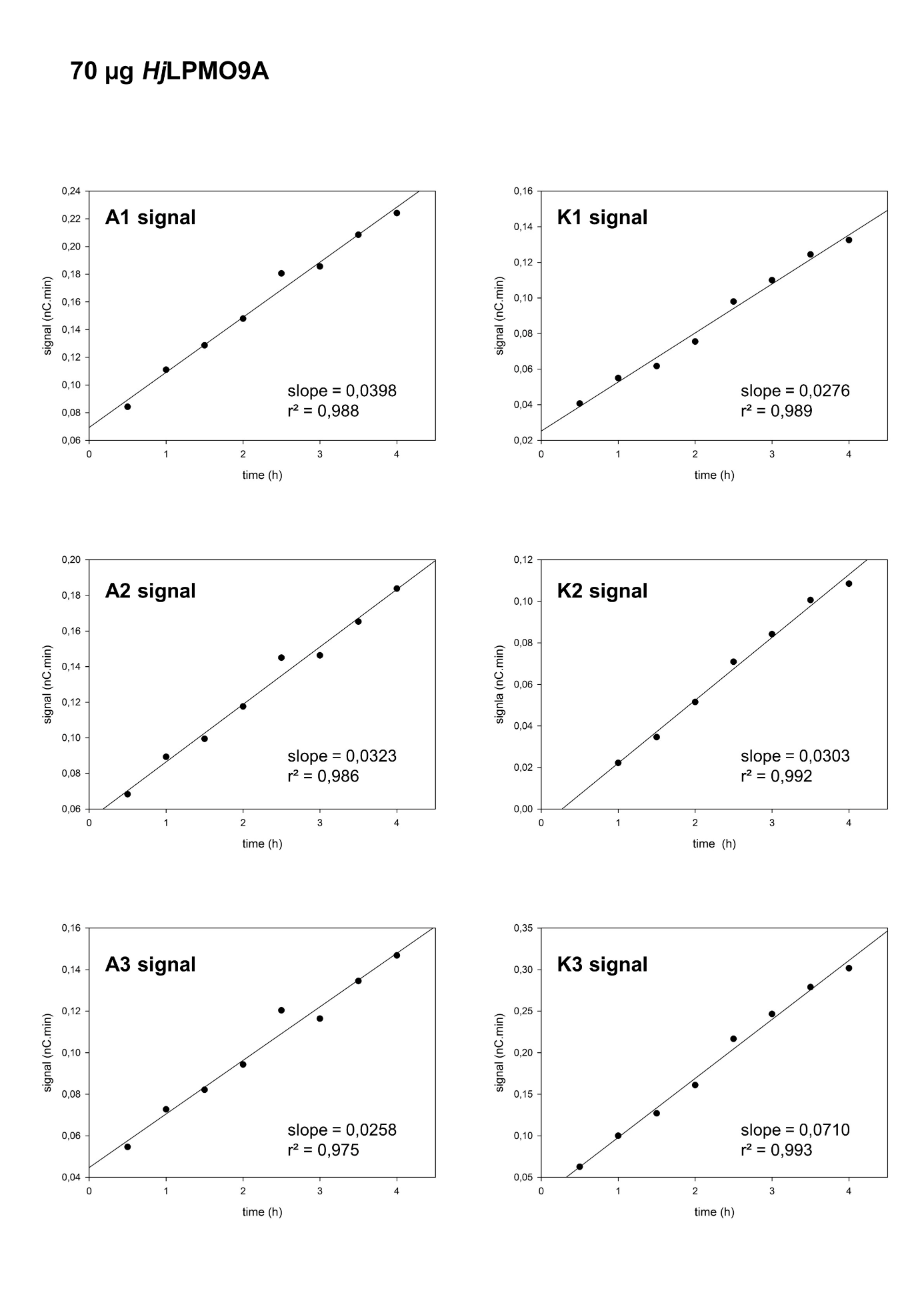


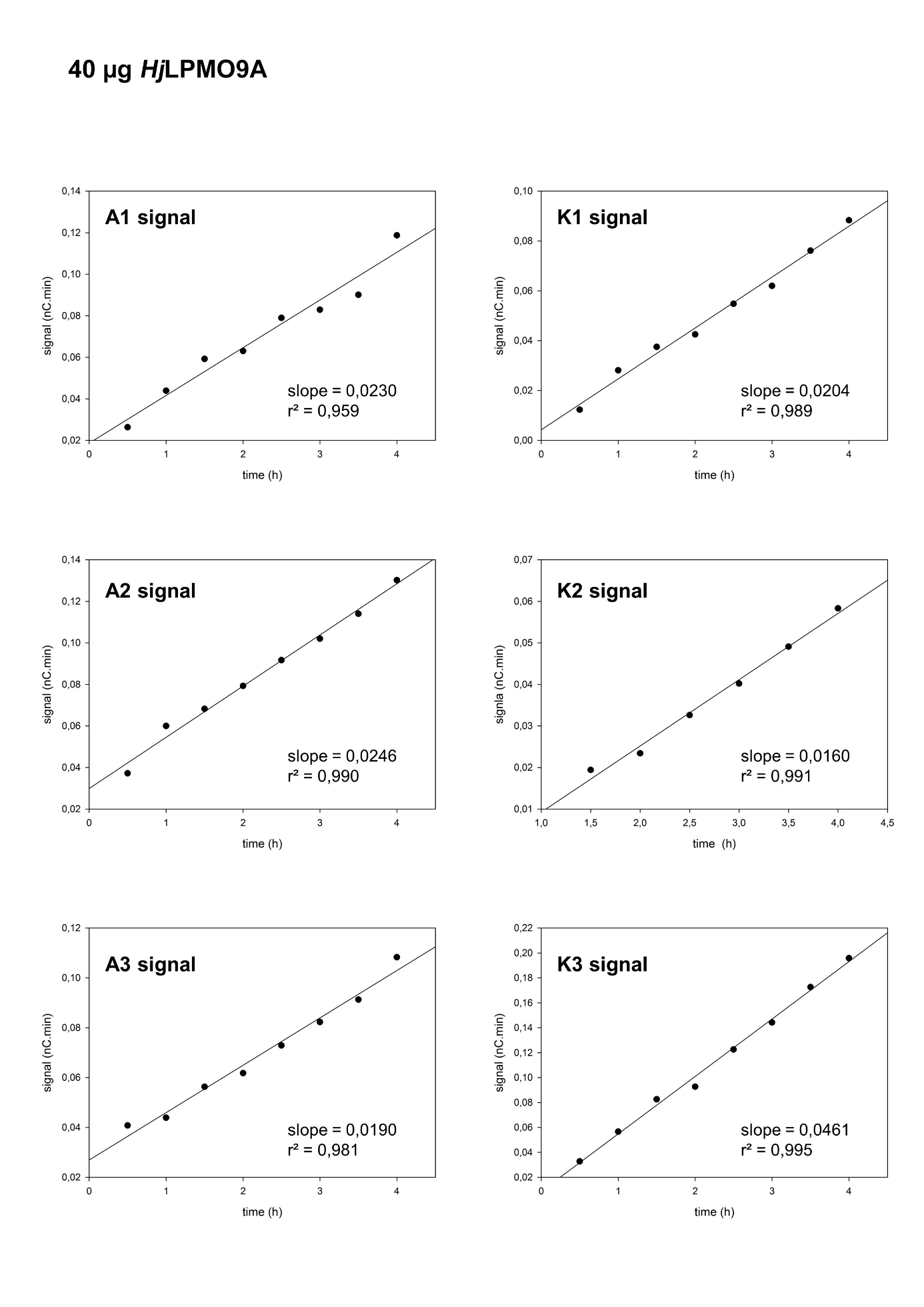


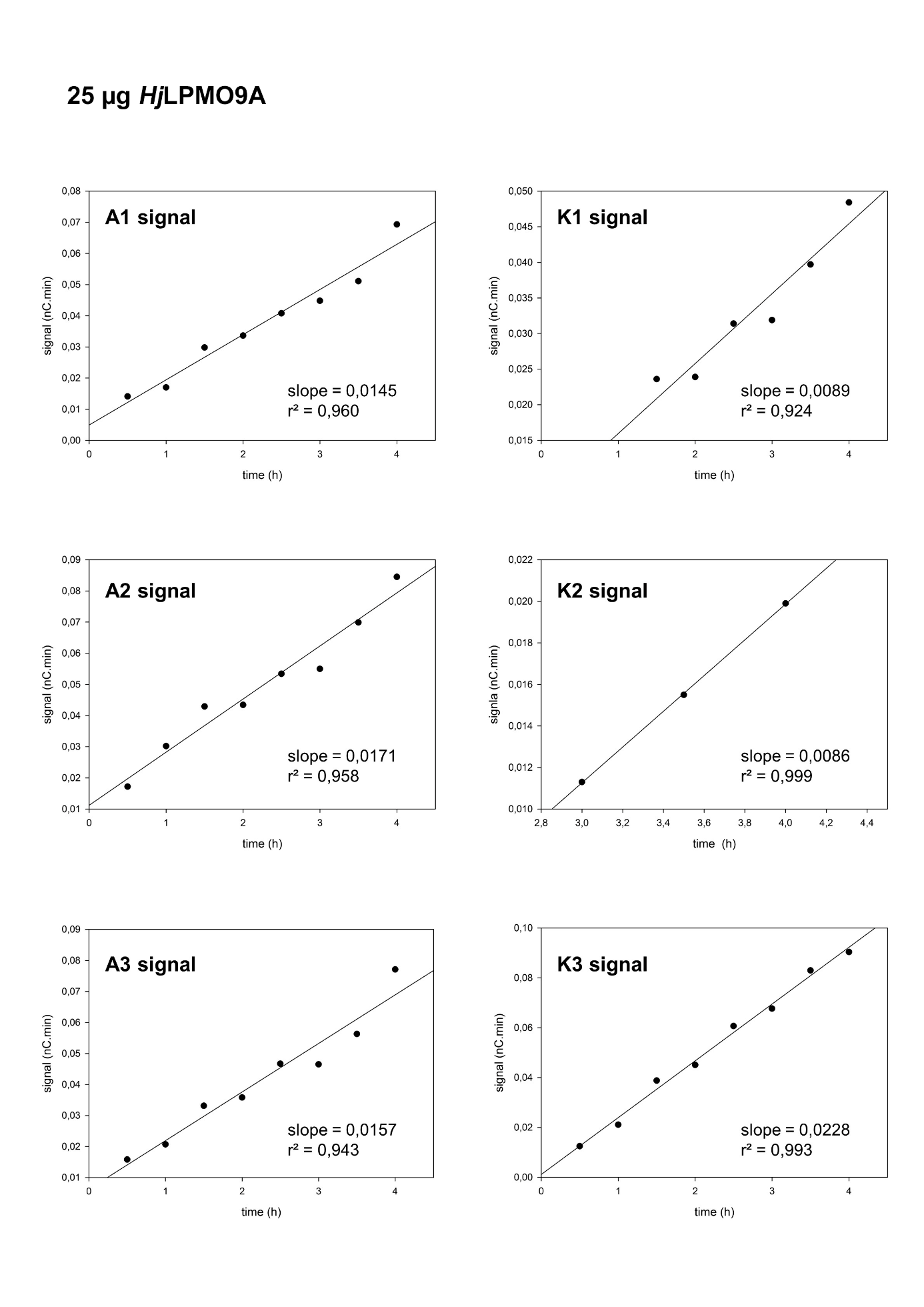


**S2 Fig. Time course monitoring of aldonic acid and 4-ketoaldose peaks.**  Time course of the three aldonic acid (A1, A2 and A3) and three 4-ketoaldose (K1, K2, K3) peaks released upon incubation of 0.5% PASC with a dilution series of *Hj*LPMO9A in 500µL enzyme tests.
