# Supplementary material for: A quantitative indicator diagram for lytic polysaccharide monooxygenases reveals the role of aromatic surface residues in HjLPMO9A regioselectivity
Source: PLoS One. 2017 May 31;12(5):e0178446. doi: 10.1371/journal.pone.0178446 (PMC5451062; doi:10.1371/journal.pone.0178446)
Supplement: S3 Fig — The total amount of oxidized cello-oligosaccharides released by 70μg of LPMO after 2.5h incubation at 50°C is compared to gain insight in the overall activity changes of the mutations. The values are means of three replicates, error bars correspond to a cumulated total standard deviation (error bar = ± total Stdev, with total Stdev = √(Stdev12 + Stdev22 + Stdev32)). (DOCX) [file pone.0178446.s003.docx]

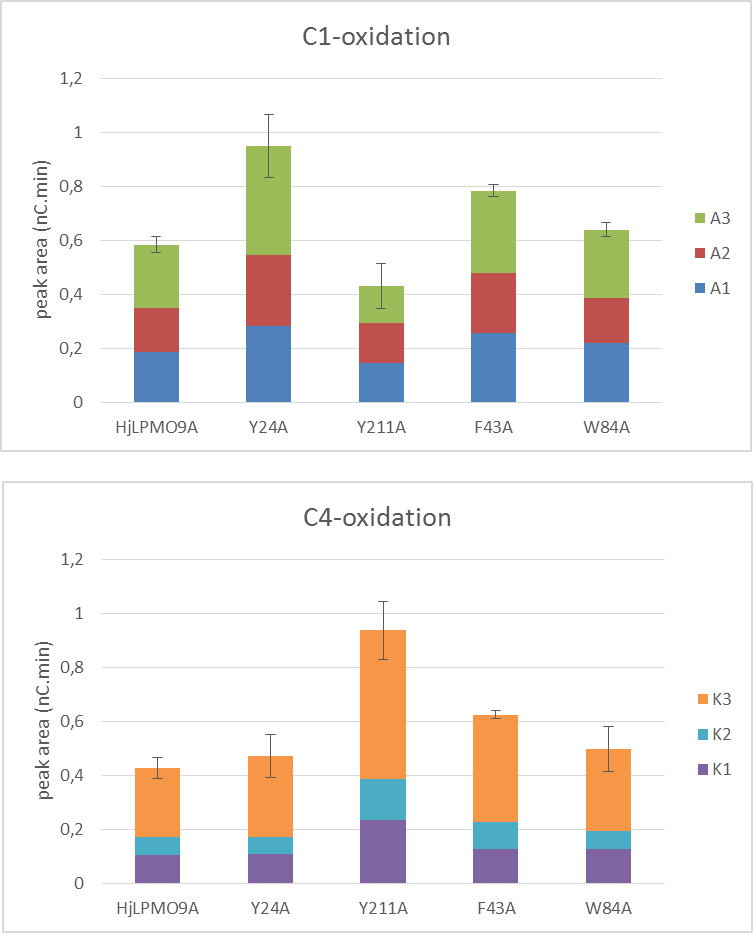


**S3 Fig. HPAEC-PAD analysis of oxidized cello-oligosaccharides released from PASC by *Hj*LPMO9A and its variants.** The total amount of oxidized cello-oligosaccharides released by 70µg of LPMO after 2.5h incubation at 50°C is compared to gain insight in the overall activity changes of the mutations. The values are means of three replicates, error bars correspond to a cumulated total standard deviation (error bar = ± total Stdev, with total Stdev = √(Stdev_1_^2^ + Stdev_2_^2^ + Stdev_3_^2^)).
