# Supplementary material for: A quantitative indicator diagram for lytic polysaccharide monooxygenases reveals the role of aromatic surface residues in HjLPMO9A regioselectivity
Source: PLoS One. 2017 May 31;12(5):e0178446. doi: 10.1371/journal.pone.0178446 (PMC5451062; doi:10.1371/journal.pone.0178446)
Supplement: S4 Fig — Chromatograms of wildtype HjLPMO9A and regioselectivity mutants Y24A (with higher C1-oxidative capacity) and Y211A (with higher C4-oxidative capacity). Two time points (after 1h and 4h incubation) are shown for each enzyme variant. (DOCX) [file pone.0178446.s004.docx]

**
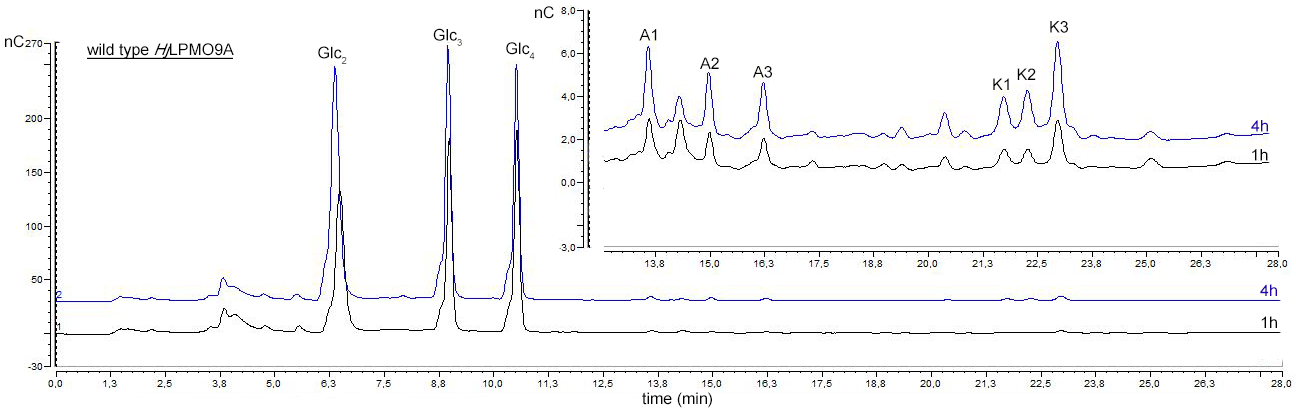
**


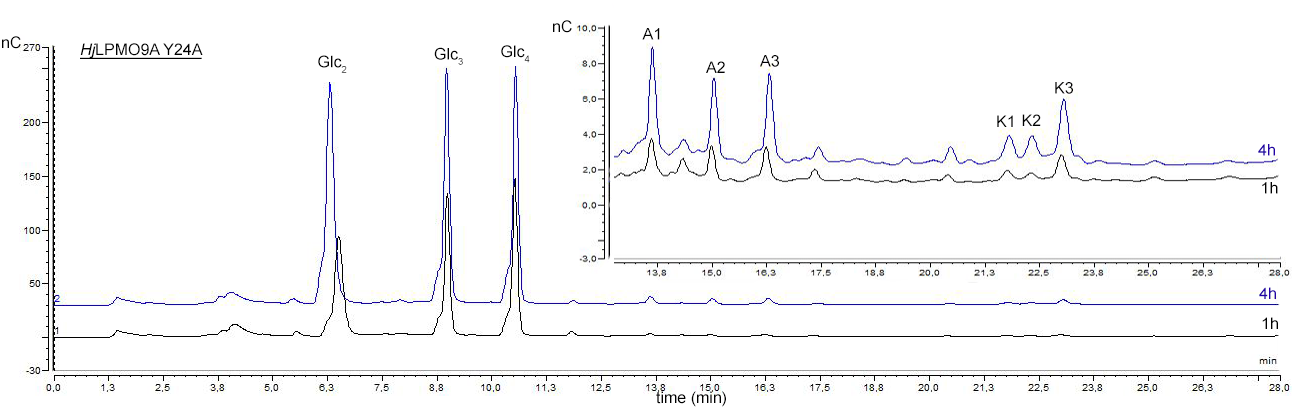


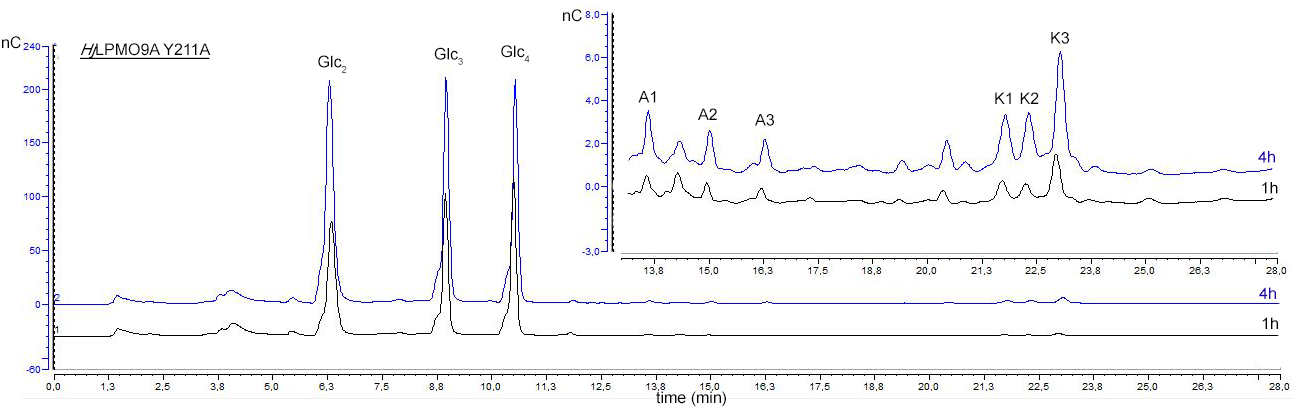


**S4 Fig.** **HPAEC-PAD chromatograms (enzyme tests with culture supernatant).** Chromatograms of wildtype *Hj*LPMO9A and regioselectivity mutants Y24A (with higher C1-oxidative capacity) and Y211A (with higher C4-oxidative capacity). Two time points (after 1h and 4h incubation) are shown for each enzyme variant.
