# Supplementary material for: A quantitative indicator diagram for lytic polysaccharide monooxygenases reveals the role of aromatic surface residues in HjLPMO9A regioselectivity
Source: PLoS One. 2017 May 31;12(5):e0178446. doi: 10.1371/journal.pone.0178446 (PMC5451062; doi:10.1371/journal.pone.0178446)
Supplement: S5 Fig — HPAEC-PAD chromatograms of wildtype HjLPMO9A and regioselectivity mutants Y24A (with higher C1-oxidative capacity) and Y211A (with higher C4-oxidative capacity) after histag purification. A control sample only containing PASC, 1mM ascorbic acid and buffer (without enzyme) was run to verify the enzyme preparations lost their endoglucanase background activity. Two time points (after 1h and 9h incubation) are shown for each enzyme variant. (DOCX) [file pone.0178446.s005.docx]

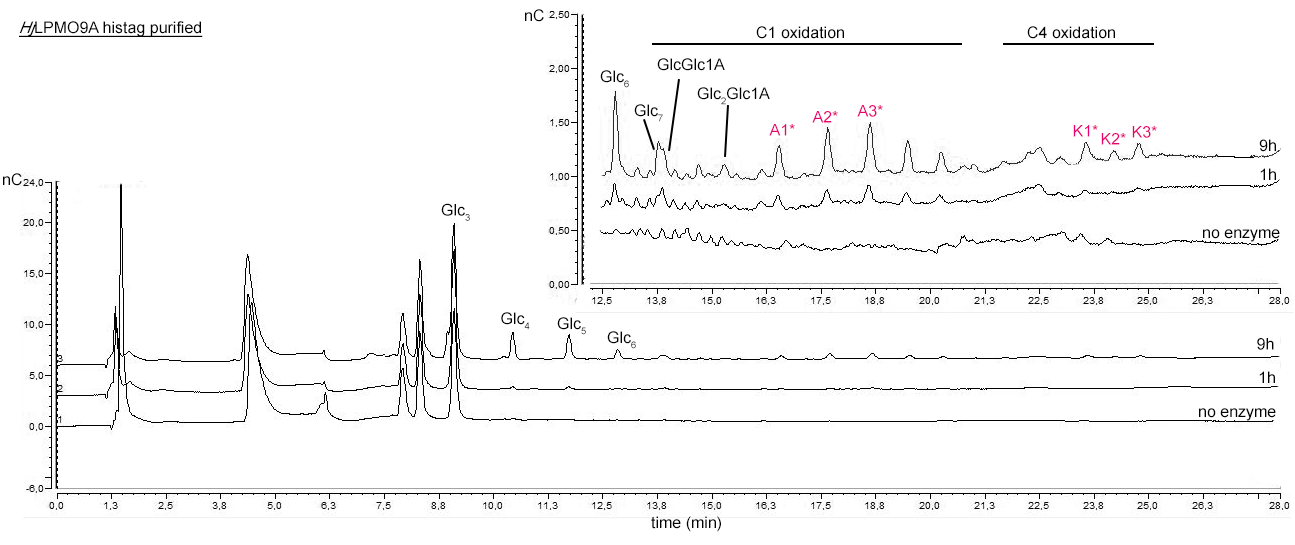


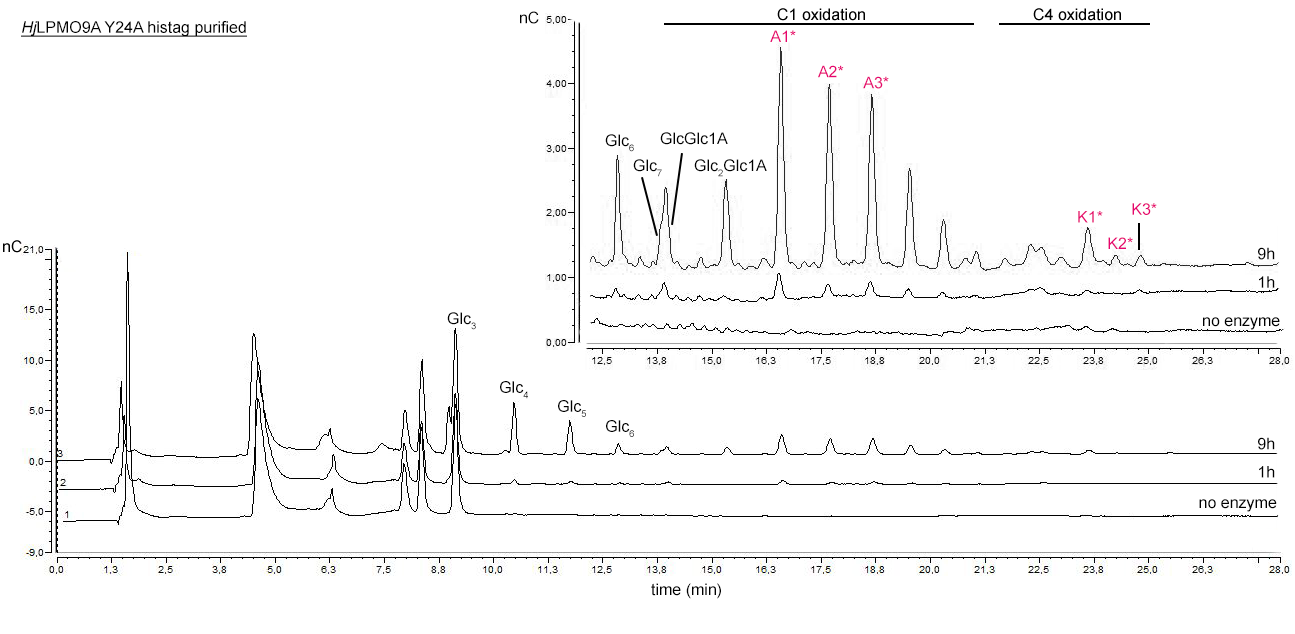


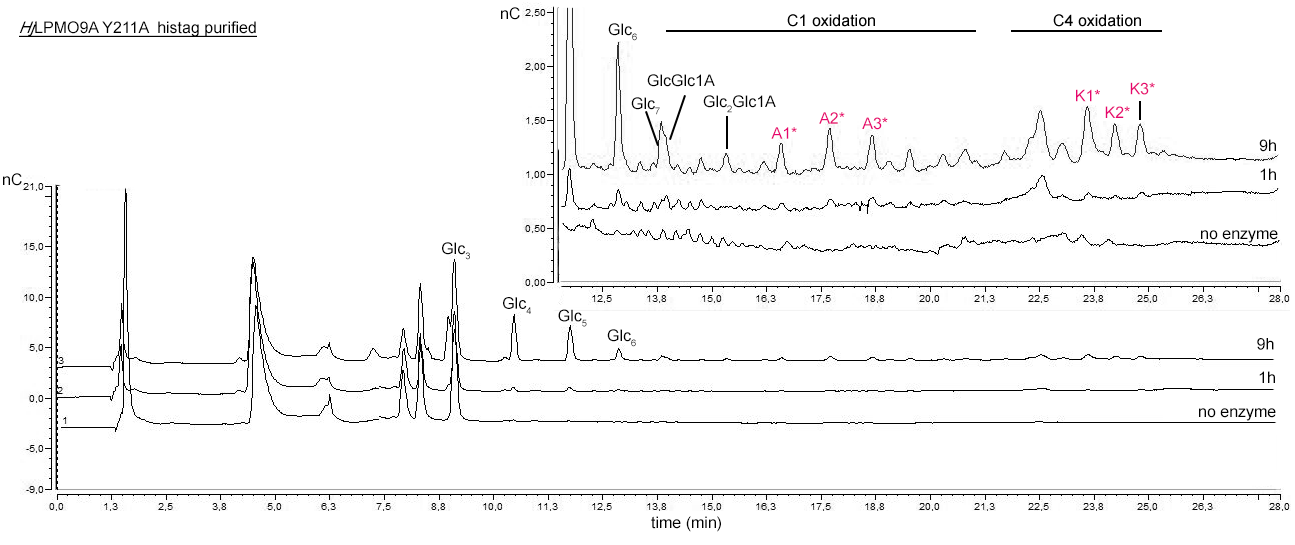


**S5 Fig.** HPAEC-PAD chromatograms of wildtype *Hj*LPMO9A and regioselectivity mutants Y24A (with higher C1-oxidative capacity) and Y211A (with higher C4-oxidative capacity) after histag purification. A control sample only containing PASC, 1mM ascorbic acid and buffer (without enzyme) was run to verify the enzyme preparations lost their endoglucanase background activity. The results histag purified broth of wildtype *P. pastoris* CBS7435 was equal to the result obtained after adding buffer (data not shown), demonstrating the purified samples lost their CDH background activity as well. Two time points (after 1h and 9h incubation) are shown for each enzyme variant.
