# Supplementary material for: A quantitative indicator diagram for lytic polysaccharide monooxygenases reveals the role of aromatic surface residues in HjLPMO9A regioselectivity
Source: PLoS One. 2017 May 31;12(5):e0178446. doi: 10.1371/journal.pone.0178446 (PMC5451062; doi:10.1371/journal.pone.0178446)
Supplement: S6 Fig — This indicator diagram was obtained for wildtype HjLPMO9A and mutants Y24A (with higher C1-oxidative capacity) and Y211A (with higher C4-oxidative capacity) after histag purification and confirms the effect on regioselectivity of the point mutations. (DOCX) [file pone.0178446.s006.docx]

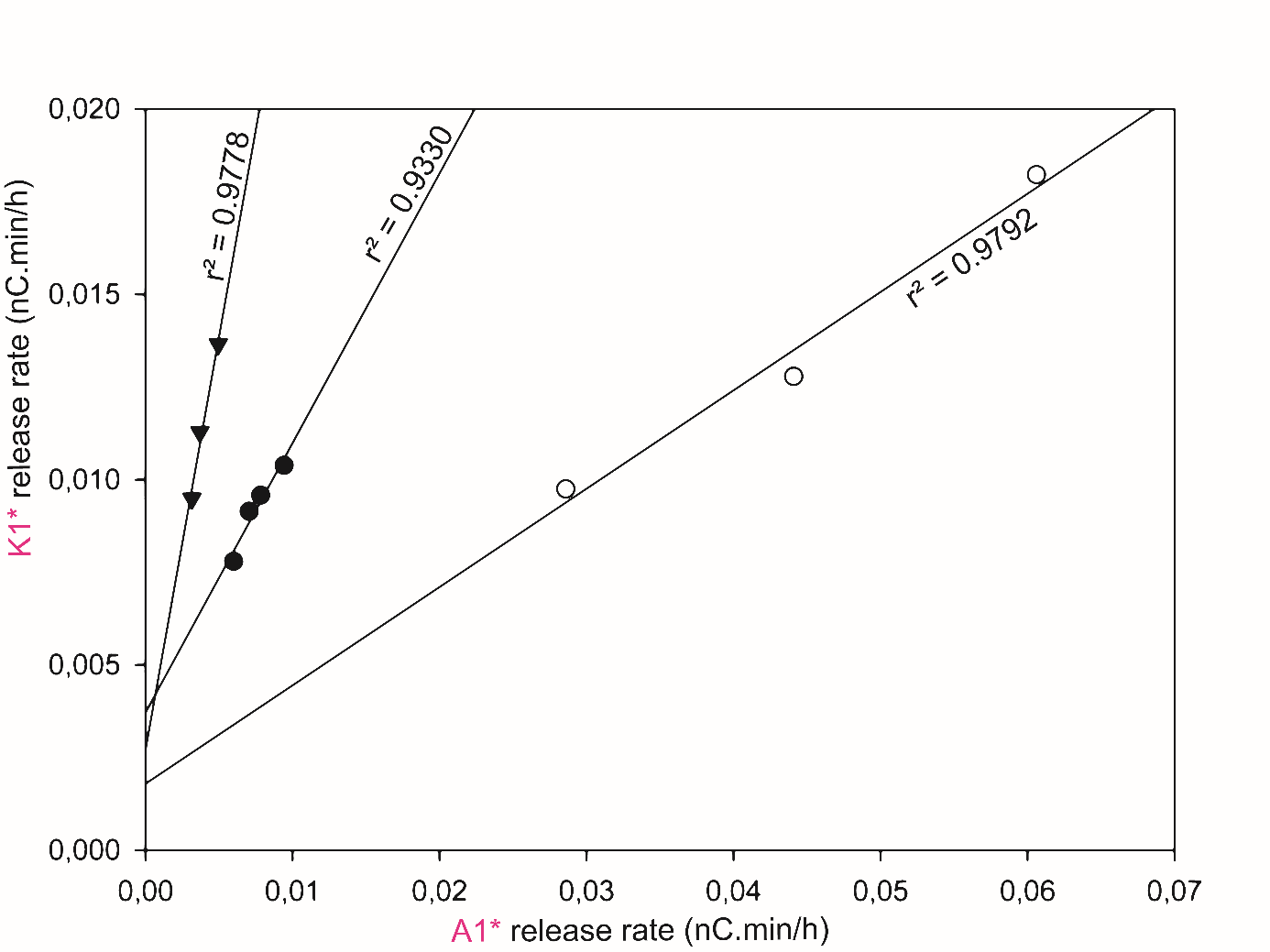


**S7 Fig.** I**ndicator diagram for purified enzymes.** This indicator diagram was obtained for wildtype *Hj*LPMO9A (●) and its mutants Y24A (○, with higher C1-oxidative capacity) and Y211A (▼, with higher C4-oxidative capacity) after histag purification and confirms the effect on regioselectivity of the point mutations. See S6 Fig for selected signals.
