# Supplementary material for: A quantitative indicator diagram for lytic polysaccharide monooxygenases reveals the role of aromatic surface residues in HjLPMO9A regioselectivity
Source: PLoS One. 2017 May 31;12(5):e0178446. doi: 10.1371/journal.pone.0178446 (PMC5451062; doi:10.1371/journal.pone.0178446)
Supplement: S1 File — (DOCX) [file pone.0178446.s007.docx]

**S1 File. Details on the C1-oxidative background activity in wildtype *P. pastoris* and its effect on the indicator diagram.**

**
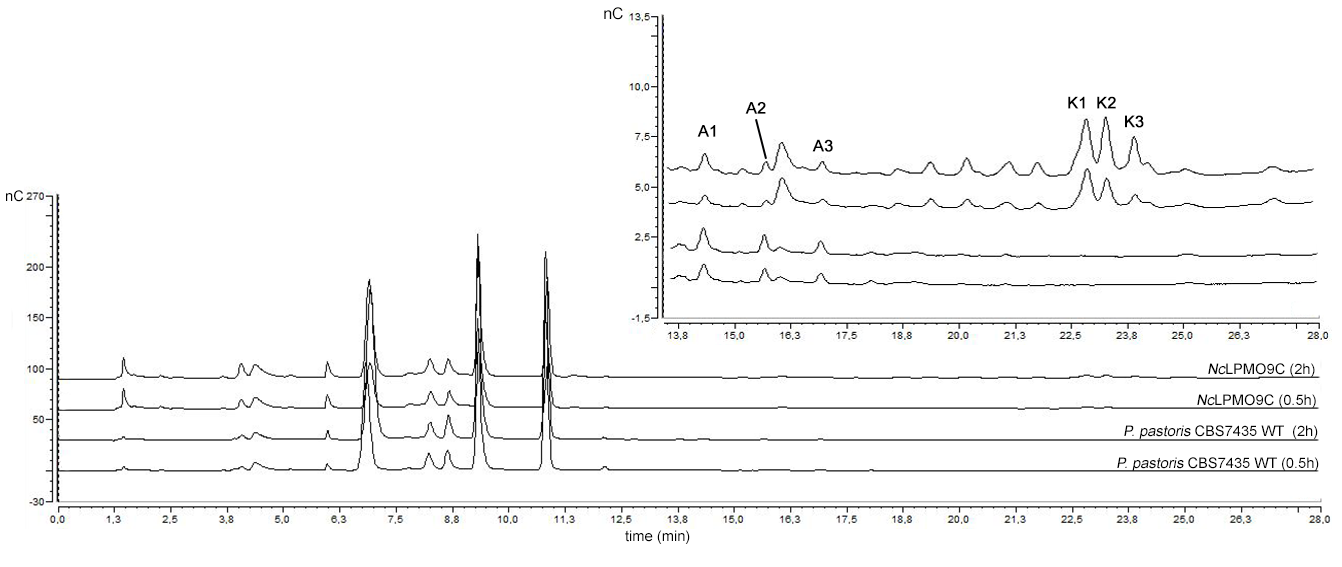
**

**Fig A**. **Traces of C1-oxidation activity in ultrafiltrated broth of *P. pastoris* CBS7435 and *Nc*LPMO9C expressing transformant.** HPAEC-PAD analysis of enzyme tests performed with 100µL ultrafiltrated culture broth. Two time points (after 0.5h and 2h) are shown.

***Nc*LPMO9C is a strict C4-oxidizing LPMO**

The minor C1-oxidative activity observed for *Nc*LPMO9C was compared to the background activity in the ultrafiltrated broth of wildtype *P. pastoris* CBS7435 (see Fig A). For this, enzyme tests were performed using 100µL ultrafiltrated broth as this corresponds to the maximal dose in the activity tests used throughout our regioselectivity study. The slope of the A2-signal formation was used as an indicator of C1-oxidation and was shown not to be significantly different for *Nc*LPMO9C when comparing to the wildtype sample (Fig B). This is in accordance with *Nc*LPMO9C being generally accepted to be a strict C4-oxidizing LPMO [1]. The results for the *P. pastoris* control strain (harboring the empty expression vector) were the same as for the wildtype *P. pastoris* CBS7435 strain.


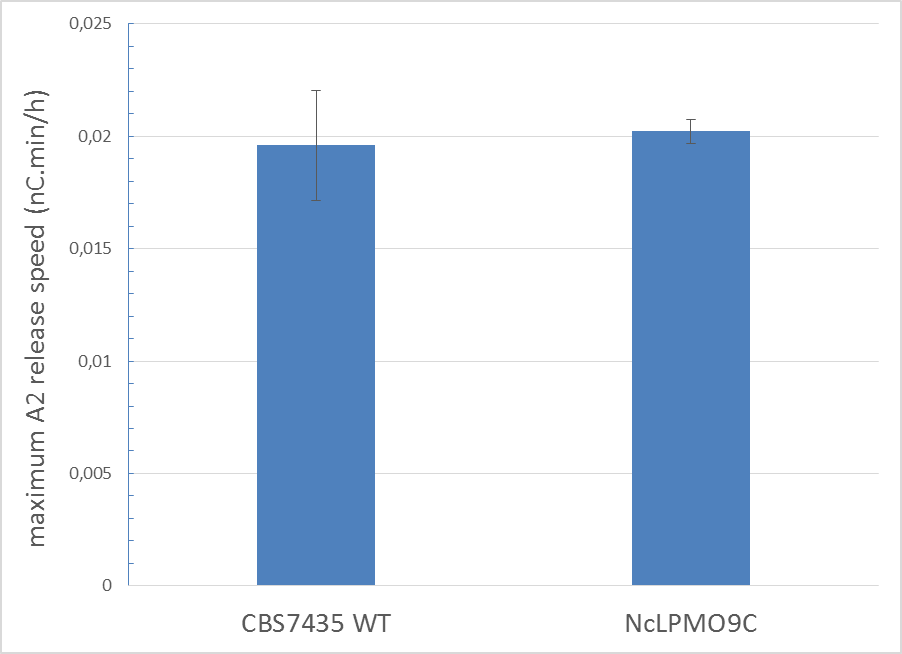


**Fig B. C1-oxidative activity in *Nc*LPMO9C broth not significantly different from that of wildtype *P. pastoris* CBS7435**. Comparison was done based on the rate of the A2 formation.

**Effect of C1-oxidation background activity on screening system**

As variants will always be compared relatively to their wildtype enzyme, the influence of the background activity is believed to be limited. This is demonstrated by the similar indicator diagram obtained for purified enzymes (see S5 Fig). Only at the lowest A2 release rates (below 0.022 nC.min/h), some prudence is called for, as it will be difficult to discriminate between background and C1-oxidative LPMO activity.

**Reference**

1. Isaksen T, Westereng B, Aachmann FL, Agger JW, Kracher D, Kittl R, et al. A C4-oxidizing lytic polysaccharide monooxygenase cleaving both cellulose and cello-oligosaccharides. J Biol Chem. 2014;289(5):2632–42.
