# Supplementary material for: A quantitative indicator diagram for lytic polysaccharide monooxygenases reveals the role of aromatic surface residues in HjLPMO9A regioselectivity
Source: PLoS One. 2017 May 31;12(5):e0178446. doi: 10.1371/journal.pone.0178446 (PMC5451062; doi:10.1371/journal.pone.0178446)
Supplement: S1 Table — Sequence of Neurospora crassa LPMO9C and Phanerochaete chrysosporium LPMO9D. (DOCX) [file pone.0178446.s008.docx]

**S1** **Table. DNA sequences.** Sequence of *Neurospora crassa* LPMO9C and *Phanerochaete chrysosporium* LPMO9D.

**> *Nc*LPMO9C native secretion signal, codon optimized for *Pichia pastoris***

1 ATGAAGACTGGTTCCATCTTGGCTGCTTTGGTTGCTTCTGCTTCCGCT

**> *Nc*LPMO9C with C-terminal his­_6_ tag, codon optimized for *P. pastoris***

1 CACACTATCTTCCAAAAGGTTTCTGTTAACGGTGCTGACCAGGGTCAGTTGAAGGGTATT

61 AGGGCTCCAGCTAACAACAACCCAGTTACTGACGTTATGTCCTCCGACATCATCTGTAAC

121 GCTGTTACTATGAAGGACTCCAACGTTTTGACTGTTCCAGCTGGTGCTAAGGTTGGTCAT

181 TTTTGGGGTCACGAAATTGGTGGTGCTGCTGGTCCAAACGATGCTGATAATCCAATTGCT

241 GCTTCCCACAAGGGTCCAATCATGGTTTACTTGGCTAAAGTTGACAACGCTGCTACTACT

301 GGTACTTCCGGTTTGAAGTGGTTCAAGGTTGCTGAAGCTGGTTTGTCCAACGGAAAGTGG

361 GCTGTTGATGACTTGATCGCTAACAACGGTTGGTCCTACTTCGACATGCCAACTTGTATT

421 GCTCCAGGTCAGTACTTGATGAGAGCTGAGTTGATCGCTTTGCACAACGCTGGTTCTCAG

481 GCTGGTGCTCAATTCTACATTGGTTGTGCTCAGATCAACGTTACTGGTGGTGGTTCTGCT

541 TCTCCATCCAACACTGTTTCTTTCCCTGGTGCTTACTCTGCTTCTGACCCAGGTATCTTG

601 ATCAACATCTACGGTGGTTCCGGTAAGACTGACAACGGTGGTAAGCCATACCAAATTCCA

661 GGTCCAGCTTTGTTCACTTGTCCTGCTGGTGGTTCAGGTGGATCTTCTCCAGCTCCTGCT

721 ACAACTGCTTCTACTCCAAAGCCAACTTCCGCTTCTGCTCCTAAGCCTGTTTCTACTACT

781 GCTTCCACACCTAAGCCTACAAACGGTTCTGGTTCTGGTACAGGTGCTGCTCACTCTACT

841 AAGTGTGGTGGATCTAAGCCAGCTGCTACAACAAAGGCTTCTAACCCACAGCCTACTAAT

901 GGTGGTAACTCCGCTGTTAGAGCTGCTGCTTTGTACGGTCAATGTGGTGGTAAAGGTTGG

961 ACTGGTCCAACTTCTTGTGCTTCCGGTACTTGTAAGTTCTCCAACGACTGGTACTCCCAG

1021 TGTTTGCCACATCACCATCACCATCACTAG

**> *Pc*LPMO9D native secretion signal, codon optimized for *Pichia pastoris***

1 ATGAAGGCTTTCTTTGCCGTTTTGGCAGTTGTCTCTGCTCCATTTGTCTTGGGT

**> *Pc*LPMO9D with C-terminal his­_6_ tag, codon optimized for *P. pastoris***

1 CACTACACTTTCCCAGACTTCATTGAGCCATCCGGTACTGTTACTGGTGACTGGGTTTAC

61 GTTAGAGAGACTCAGAACCACTACTCCAACGGTCCAGTTACTGACGTTACTTCCCCAGAG

121 TTCAGATGTTACGAGTTGGACTTGCAGAACACTGCTGGTCAAACTCAGACTGCTACTGTT

181 TCTGCTGGTGACACTGTTGGTTTCAAGGCTAACTCTGCTATCTACCACCCAGGTTACTTG

241 GACGTTATGATGTCTCCAGCTTCTCCTGCTGCTAACTCTCCAGAAGCTGGTACTGGACAG

301 ACATGGTTCAAGATCTACGAAGAGAAGCCACAGTTCGAGAACGGTCAGTTGGTTTTCGAC

361 ACTACTCAGCAAGAGGTTACTTTCACTATCCCAAAGTCCTTGCCTTCCGGTCAGTACTTG

421 TTGAGAATTGAGCAGATCGCTTTGCACGTTGCTTCATCTTACGGTGGTGCTCAGTTCTAC

481 ATTGGTTGTGCTCAATTGAACGTTGAGAACGGTGGTAACGGTACTCCAGGTCCATTGGTT

541 TCTATCCCAGGTGTTTACACTGGTTACGAGCCAGGTATCTTGATCAACATCTACAACTTG

601 CCAAAGAACTTCACTGGATACCCAGCTCCAGGACCAGCTGTTTGGCAAGGTGAACAAAAA

661 CTCATCTCAGAAGAGGATCTGAATAGCGCCGTCGACCATCATCATCATCATCATTGA
